# Inter- and intra-host sequence diversity reveal the emergence of viral variants during an overwintering epidemic caused by dengue virus serotype 2 in southern Taiwan

**DOI:** 10.1371/journal.pntd.0006827

**Published:** 2018-10-04

**Authors:** Hui-Ying Ko, Yao-Tsun Li, Day-Yu Chao, Yun-Cheng Chang, Zheng-Rong T. Li, Melody Wang, Chuan-Liang Kao, Tzai-Hung Wen, Pei-Yun Shu, Gwong-Jen J. Chang, Chwan-Chuen King

**Affiliations:** 1 Institute of Epidemiology and Preventive Medicine, College of Public Health, National Taiwan University, Taipei, Taiwan, Republic of China; 2 School of Veterinary Medicine, Institute of Microbiology and Public Health, National Chung-Hsing University, Taichung, Taiwan, Republic of China; 3 Department of Clinical Laboratory Sciences and Medical Biotechnology, College of Medicine, National Taiwan University, Taipei, Taiwan, Republic of China; 4 Department of Geography, College of Science, National Taiwan University, Taipei, Taiwan, Republic of China; 5 Center for Diagnostics and Vaccine Development, Centers for Disease Control, Ministry of Health and Welfare, Taiwan, Republic of China; 6 Arboviral Diseases Branch, Division of Vector-Borne Diseases, Centers for Disease Control and Prevention, Fort Collins, Colorado, United States of America; Oregon Health and Science University, UNITED STATES

## Abstract

Purifying selection during dengue viral infection has been suggested as the driving force of viral evolution and the higher complexity of the intra-host quasi-species is thought to offer an adaptive advantage for arboviruses as they cycle between arthropod and vertebrate hosts. However, very few studies have been performed to investigate the viral genetic changes within (intra-host) and between (inter-host) humans in a spatio-temporal scale. Viruses of different serotypes from various countries imported to Taiwan cause annual outbreaks. During 2001–2003, two consecutive outbreaks were caused by dengue virus serotype 2 (DENV-2) and resulted in a larger-scale epidemic with more severe dengue cases in the following year. Phylogenetic analyses showed that the viruses from both events were similar and related to the 2001 DENV-2 isolate from the Philippines. We comprehensively analyzed viral sequences from representative dengue patients and identified three consensus genetic variants, group Ia, Ib and II, with different spatio-temporal population dynamics. The phylodynamic analysis suggested group Ib variants, characterized by lower genetic diversity, transmission rate, and intra-host variant numbers, might play the role of maintenance variants. The residential locations among the patients infected by group Ib variants were in the outer rim of case clusters throughout the 2001–2003 period whereas group Ia and II variants were located in the centers of case clusters, suggesting that group Ib viruses might serve as “sheltered overwintering” variants in an undefined ecological niche. Further deep sequencing of the viral envelope (E) gene directly from individual patient serum samples confirmed the emergence of variants belonging to three quasi-species (group Ia, Ib, and II) and the ancestral role of the viral variants in the latter phase of the 2001 outbreak contributed to the later, larger-scale epidemic beginning in 2002. These findings enhanced our understanding of increasing epidemic severity over time in the same epidemic area. It also highlights the importance of combining phylodynamic and deep sequencing analysis as surveillance tools for detecting dynamic changes in viral variants, particularly searching for and monitoring any specific viral subpopulation. Such subpopulations might have selection advantages in both fitness and transmissibility leading to increased epidemic severity.

## Author summary

Virus genomes can show changes due to transmission between hosts. In evolutionary scale, replacements with new viral lineages are usually associated with alternating clinical burdens or epidemic potentials, but few studies have integrated viral dynamics from intra-host viral variations to macro-level clade (a monophyletic group) replacements. In this study, we combined both levels of research to study the changes in viral genomes during two consecutive dengue outbreaks (2001–2003) in southern Taiwan. We identified a potential overwintering candidate virus group characterized by lower genetic diversity, transmission rate, number of intra-host variants and a more pervasive geographical distribution pattern. Deep sequencing data revealed the temporal dynamics of minor variants in different nucleotide positions, correlated with the phylogenetic analyses of consensus sequences derived from different patients. We thus propose that investigating the quasi-species complexity of viruses from intra-host and inter-host infections can provide a novel understanding of virus evolution. Such investigations could also be a useful method to monitor the dynamics of viral changes and be included in the virological surveillance.

## Introduction

Dengue fever (DF) is the most widely distributed and rapidly spread vector-borne viral disease, transmitted among human populations mainly through the biting of female *Aedes* mosquitos [1]. Currently, an estimated 2.5 billion people are at risk for virus infection and 390 million dengue virus (DENV) infections occur annually [2]. The World Health Organization in 2015 predicted that dengue infections would continue to increase due to global warming and frequent international travel [2–4]. Most people infected with DENV are either asymptomatic or have mild symptoms of dengue fever (DF). Only about 6% of dengue patients present with severe dengue hemorrhagic fever (DHF) or dengue shock syndrome (DSS). Although there is no specific treatment for dengue, case fatality rates can be approximately 1% with proper case management [5].

DENV includes four serotypes, designated as DENV-1 to 4, and belong to the genus *Flavivirus* in the *Flaviviridae* family. The viral genome is approximately 10.7 kilobases (kb) in length and contains a single open reading frame (ORF) encoding three structural proteins: capsid (C), precursor membrane/membrane (prM/M) and envelope (E) proteins, and seven non-structural proteins: NS1, NS2A, NS2B, NS3, NS4A, NS4B and NS5, flanked by 5’ and 3’ untranslated regions (UTR) [6]. Increased epidemic scale and disease severity have been associated with accumulation of genetic changes scattered throughout the entire viral RNA genome in circulating viral strains. Consequently, multiple genotypes, strains or variants within a serotype can co-circulate in the same geographical area. However, the viral population dynamics are complex, involving both the emergence and the death of viral lineages that may differ in transmissibility, virulence, and fitness as well as the intricate patterns of gene flow within and between the alternating hosts of human and mosquito [7–11].

**Fig 1:**
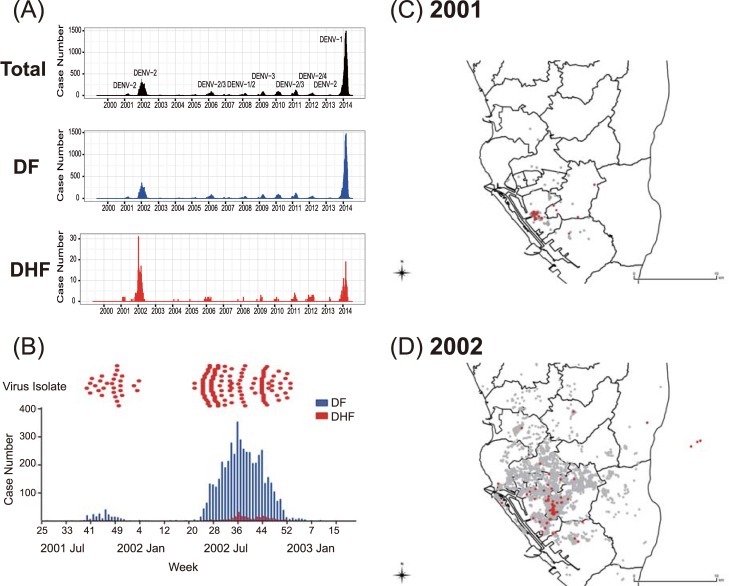
Spatio-temporal distributions of DENV-2 isolated and sequenced from patients in Taiwan, during 2001–2003. (A) The number of laboratory-confirmed, indigenous dengue cases and their major viral serotype found in Kaohsiung city from January 2000 to April 2015. The total numbers of laboratory-confirmed dengue cases, cases diagnosed with dengue fever (DF, blue) or dengue hemorrhagic fever (DHF, red) are shown separately. (B) The isolated DENV-2 strains at each of the respective time points (upper panel), compared to the total numbers of confirmed dengue cases (5305, including 5064 DF and 241 DHF cases) embedded in the epidemic curve for the same period (lower panel). Two consecutive outbreaks occurred in the period from the 35th week, 2001 to the 11th week, 2003: 232 confirmed cases, including 222 DF and 10 DHF cases in 2001; and 5073 confirmed cases, including 4842 DF and 231 DHF cases. (C)(D) Spatial distributions of dengue cases in 2001 (C) and 2002 (D). Red dots represent viral isolates that were investigated in this study; gray plot represents dengue cases reported from Taiwan-CDC.

Dengue is not endemic in Taiwan, unlike other endemic areas in Southeast Asia with multiple serotypes co-circulating at the same time (Fig 1A). Surveillance data from the Taiwan Centers of Disease Control (Taiwan-CDC) supports the premise that dengue viruses are imported into Taiwan mainly from Southeast Asia with 75% of imported cases from Vietnam, Indonesia, the Philippines, and Thailand [12]. Continuous introduction of viruses by dengue viremic travelers has led to annual local outbreaks with one dominant serotype, mainly occurring in southern Taiwan, where *Aedes aegypti mosquitoes* are the main vector in urban areas. The outbreaks usually terminate in the winter when mosquito abundance declines [13]. However, during the years of 2001–2003, two consecutive outbreaks caused by DENV-2 occurred in Kaohsiung City in southern Taiwan, which resulted in 5,311 DF and 252 DHF/DSS cases, and 21 deaths. The epi-curve showed that case numbers in 2002 surpassed those in the 2001 outbreak (5,336 vs. 227, respectively, Fig 1B). These overwintering outbreaks provided us with a great opportunity to study the viral population dynamics during the two consecutive outbreaks, particularly the changes during the winter.

In this study, we tracked the viral phylodynamic changes between (inter-host) and within dengue patients (intra-host) over different times and areas, based on the complete ORF and E gene sequences. Three quasi-species variants (Ia, Ib, and II) were identified. Each variant had a different spatio-temporal viral population dispersion pattern. Group Ib was a minor population persistently circulating through the epidemic with low genetic diversity, transmission rate and number of intra-host variants confirmed by deep sequencing. This study highlighted that the combination of phylodynamic and deep sequencing analysis can be an important surveillance tool for detecting dynamic changes in viral population diversity and expansion, as well as characterizing differences in fitness and transmissibility of a particular dominant variant sub-population arising by the selection through an epidemic process.

## Materials and methods

### Study area

The study areas are 12 Districts of Kaohsiung City (including 11 Districts from the old Kaohsiung administrative Districts and Fengshan District) and Pingtung City, the capital of Pingtung County, the two cities located in southern Taiwan with a tropical climate suitable for *Aedes aegypti* and *Aedes albopictus* mosquitoes to transmit DENV [14]. Kaohsiung City (the second largest metropolis in Taiwan) and its neighboring Fengshan District (an extended area of Kaohsiung City) had a population density of about 10,200 people/km^2^ in 2002. In Taiwan, the District is composed by “Li” (equivalent to the village), the basic administrative unit in Taiwan, thus we used “Li” as the spatial unit as in our previous studies [15, 16]. Kaohsiung has both a seaport and an airport for international travelers who may bring DENV into the city whereas Pingtung City (population of 215,584 persons in 2002 covering 65.067 square kilometers) is an older city with many old buildings providing excellent mosquito breeding habitats. Both Kaohsiung and Pingtung also have a higher percentage of foreign laborers from dengue-endemic countries as well as a high population density able to maintain viral transmission locally. Dengue outbreaks in these two study areas occur when two conditions are met: the introduction of the virus to the general population by dengue viremic travelers and appropriate meteorological factors favoring *Aedes* breeding [17]. Failure to implement mosquito control permitting virus circulation occasionally allows DENV over-wintering to occur. Compared with dengue-endemic countries, Taiwan is unique in having had only one overwintering serotype of DENV between 2001 and 2002. This situation offered the best chance to investigate DENV dynamics during two consecutive over-wintering epidemics in Kaohsiung and Pingtung.

### Study participants and data source

From September 2001 through March 2003, laboratory-confirmed dengue cases from Kaohsiung and Pingtung areas accounted for 90.4% (5062/5602) of total dengue cases in Taiwan, were included in this study. Cases, reviewed by Dengue Clinical Committee in Taiwan, were classified as DF or DHF based on the clinical guidelines published by World Health Organization-1997 [18]. Laboratory diagnosis of current DENV infection was based on dengue virus-specific immunoglobulin M (IgM) and IgG antibody-capture enzyme-linked immunosorbent assay [19], reverse transcriptase-polymerase chain reaction (RT-PCR), or virus isolation in cell cultures [20, 21]. The study was approved by the Institute Review Board (IRB) of the hospitals participating in this study, mainly Yuan General Hospital, Huei-Te Hospital and Pingtung Christian Hospital and the IRB of National Taiwan University Hospital (NTUH-REC No. 200903086R) as described in a previous study [22]. The blood samples were collected from children under age 18 with approved written consent from guardians and adults with approved written consent. Plasma was separated and stored at -80°C. A total of 2,234 blood samples (some included paired or triple specimens from the same individual) were collected from 1,565 dengue-confirmed patients during the study period. Secondary DENV infections were determined by dengue-specific IgM and IgG capture ELISA (Inbios DENV Detect IgM Capture ELISA kit and DENV Detect^TM^ IgG Capture ELISA kit) performed as previously described [20].

### Virus isolation and sequencing

Plasma from the patients’ acute phase specimens (within 5 days post onset of fever) collected in 2002 was selected according to defined temporal and spatial characteristics, whereas all the 2001 samples were included in this study (Fig 1B–1D). DENV-RT-PCR-positive patient’s plasma was inoculated onto C6/36 cells derived from *Aedes albopictus* mosquitoes (American Type Culture Collection (ATCC)) and grown at 28°C in 10% fetal bovine serum (FBS, ThermoFisher) containing Mitsuhashi-Maramorosch medium (HiMedia) plus Dulbecco's modified Eagle's medium (ThermoFisher) and 1% penicillin/streptomycin (ThermoFisher). For virus isolation, the patient’s plasma was inoculated onto C6/36 cells and maintained in the same media with 2% FBS. At 5–10 days after inoculation, the culture supernatant was harvested when C6/36 cells showed more than 50% cytopathic effect (CPE). RT-PCR was used to confirm the presence of virus in the C6/36 cultured supernatant. Forty-three DENV-2 isolates from C6/36 cells, stored at -80°C, were used to obtain the consensus sequence of the complete ORF in this study (methods as described below).

Dengue viral RNA was extracted directly from patient’s plasma or RT-PCR confirmed C6/36 cultured supernatant using a QIAmp viral RNA mini kit (Qiagen). cDNA was synthesized by SuperScript III Reverse Transcriptase kit (ThermoFisher), using random hexamers (Promega). Virus-specific PCR was performed using a Platinum Taq DNA Polymerase Kit (ThermoFisher) and consensus sequences were obtained using the conventional Sanger sequencing method [23]. Primers used to amplify ten overlapping PCR fragments were listed in the supplementary table (S1 Table). Four primers, d2-518F, d2E+34B, d2-E420F, d2-E712R, were used to obtain complete viral E protein sequences.

### Phylogenetic and genetic analyses

Time-scaled phylogenies were inferred by the Bayesian Markov Chain Monte Carlo (MCMC) method using BEAST v1.82 [24]. A total of 129 complete viral E genes, including 104 DENV-2 sequences from RT-PCR confirmed cases obtained in this study and 25 reference sequences retrieved from GenBank, were used to construct a maximum clade credibility (MCC) tree [25]. S2 Table lists the accession numbers of all sequences included in this study. Complete ORF sequences (10176 nt.) of 43 C6/36 isolated DENV-2 viruses were also subjected to an MCC tree construction. The best-fit DNA substitution model was determined by Akaike Information Criterion (AIC) and Bayesian Information Criterion (BIC) implemented in jModelTest [26, 27]. Both AIC and BIC indicated that the TN93+G model was the best fit for the dataset, and a strict molecular clock was applied. S3 Table lists log marginal likelihoods of various models by different methods. Two*10^8^ and 1*10^7^ MCMC iterations were implemented for E protein and complete ORF, respectively. Tracer v1.8 (available in BEAST package) was used to ascertain the calibration and ensure the effective sample sizes (ESS) of higher than 200 for all parameters. MCC trees were generated by TreeAnnotator v1.8.1 (available in BEAST) after removing 10% as burn-ins, visualized and summarized by FigTree v1.4.2 [28] with posterior probabilities. The viral group evolutionary rates were calculated using Tracer v1.8 after being combined by LogCombiner v1.8.4 (available in BEAST) with constant population size as a tree priority. To obtain root-to-tip genetic distances, maximum likelihood phylogenetic trees were constructed with DENV-2 E and ORF genes using MEGA 6.06 software [29]. The genetic distances were plotted against time by R v3.30 [30]. Phylogenetic trees were visualized with R package ggtree [31].

### Spatio-temporal analyses

We analyzed geo-coding information about dengue cases by QGIS 2.0 from previous studies [32, 33]. We generated matrices of distance between the samples to examine virus movement. The matrices of distance showed the geographic relatedness of each sample, considering their dates and places of collections and viral genetic sequences. GEO SPHERE package in BEAST v2.2 was applied to implement Bayesian phylogeographical analysis using a diffusion on a sphere model [34]. Bayesian MCMC analyses using the HKY+G nucleotide substitution model with a strict molecular clock were run for 10^9^ steps for E genes of group Ia and Ib virus variants, and 9*10^8^ steps for group II virus variants. The direction of virus movement was labeled based on the locations of the most recent common ancestor (MRCAs) inferred from the MCC trees by QGIS.

### Estimation of effective reproductive number (R)

We used the birth-death skyline model, implemented in the BEAST 2.2 birth-death skyline serial package, to estimate the transmissibility of viral variants [35]. The birth-death skyline serial analysis describes a birth-death process, assuming that each infected individual might transmit with a rate λ and eventually becomes noninfectious with a rate δ. An individual virus was sampled with a probability ρ and included in the dataset. We used the DNA substitution model HKY+G with a strict molecular clock and Gamma distribution. The iteration numbers included 5*10^7^ MCMC for group Ia variants and at least 9.5*10^8^ for groups Ib and II variants to ensure ESS higher than 200. The R values of the three viral variant groups were estimated with 95% highest posterior density (HPD) intervals. The sampling rate was set at 0.29 (1,565 cases were included in this study among 5,446 confirmed dengue cases).

### Deep sequencing of viral E-gene populations

Virus-specific cDNAs flanking the E gene region from 77 plasma samples were deep sequenced and analyzed to estimate and compare the complexity of viral populations (quasi-species; noted in S2 Table and listed in S6 Table). Briefly, viral RNA was extracted from 140 μl of plasma and cDNA synthesized following the procedure described in the “Virus isolation and sequencing” section above. One μl of cDNA from each sample was subjected to PCR using Phusion High-Fidelity DNA Polymerase (ThermoFisher) with primer sets targeting the E protein gene to construct the cDNA library. Primer sets were designed to amplify the E protein gene along with Illumina overhang adapter (S4 Table). Four overlapping fragments of 400–500 bp were first amplified by 25 PCR cycles. These products were then amplified by 15 additional cycles using sample-specific Illumina dual i5 and i7 index adapters to label each sample. The resulting amplicons were purified using AMPure XP beads (ThermoFisher) and further quantified using a Qubit 3.0 NGS Starter Kit (ThermoFisher). The purified amplicons were then clustered and sequenced with Illumina MiSeq Platform (Illumina) at Technology Commons, College of Life Science, National Taiwan University. For quality control, sequences were removed from analysis if quality scores (Phred) were below 30 using CLC Genomics Workbench 6.0 software (CLC Bio). All sequences were mapped based on consensus sequences of the 2001 DENV-2 Kaohsiung isolates and adaptor sequences were removed using CLC. Sequence files were analyzed using program R. LoFreq was used to detect minor viral variants to study the intra-host heterogeneity of viral variants (quasi-species). LoFreq models sequencing error rate and implements a Poisson distribution to probe the statistical significance of nucleotide variants at each position [36]. To eliminate primer bias, variant(s) detected in the primer region were verified by sequences obtained from the reverse primer. A position was considered to be a variant site when the coverage depth was higher than 1000.

### Nucleotide sequence accession numbers

Consensus genome assemblies and annotations generated as part of this project were submitted to GenBank with the following accession numbers: MG457038- MG457070 and MG599558—MG599634.

## Results

### Phylogenetic relationships among the three groups of DENV-2 virus strains isolated from the two consecutive dengue outbreaks (2001–2003) in Kaohsiung

**Fig 2:**
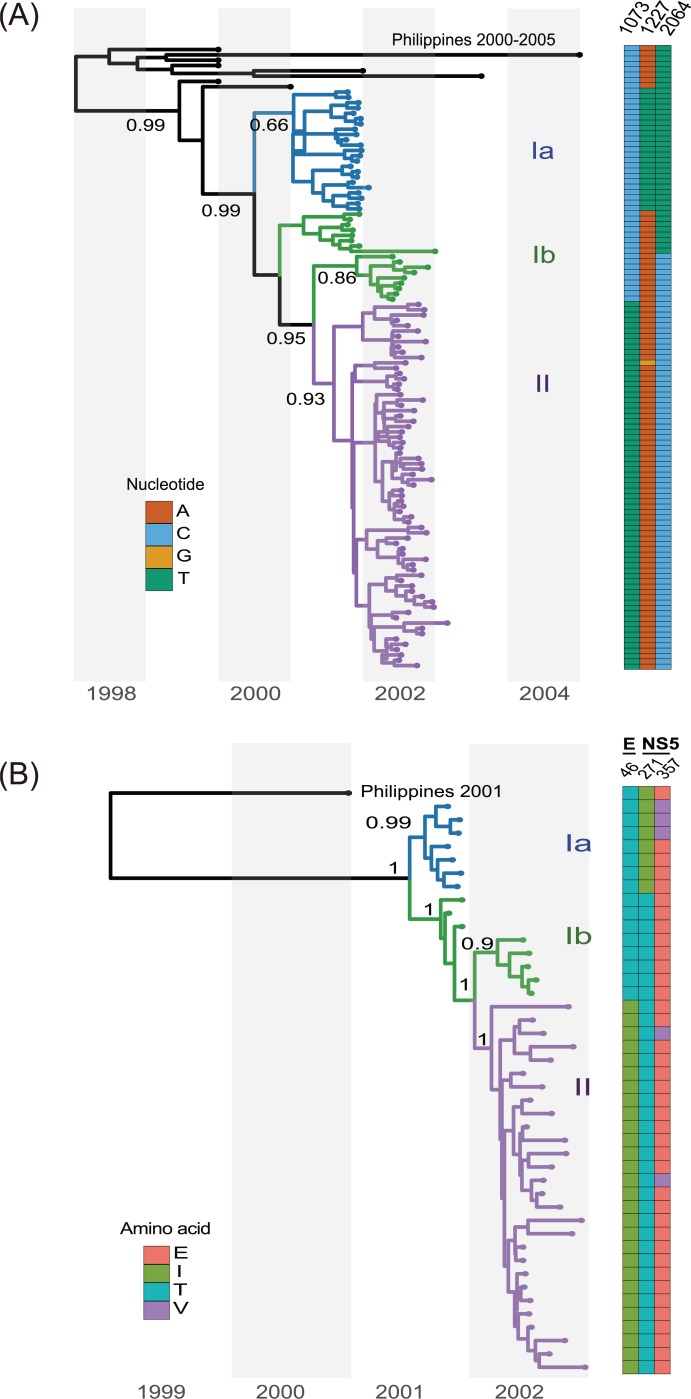
Maximum clade credibility (MCC) tree of the E and ORF genes. (A) A MCC tree based on the E genes of 104 DENV-2 viruses isolated during 2001–2003 in southern Taiwan together with those from the Philippines as a reference group. Based on the tree and nucleotide signatures, group I [nt-1073C; threonine (T) at E-46] and group II [nt-1073T; isoleucine (I) at E-46] were identified. Posterior probabilities were labeled on distinct nodes. Heatmap next to the tree indicates three corresponding nucleotide positions in the E gene for each of the different isolates. (B) MCC tree based on ORF of 43 Taiwan DENV-2 isolates. Two distinguishable virus variants were further noted in group I viruses: Ia viruses have isoleucine (I) at the NS5-271 position and valine/glutamate (V/E) at the NS5-357 position, whereas Ib viruses have threonine (T) at NS5-271 and glutamate (E) at NS5-375.

Phylogenetic analyses using the maximum likelihood (ML) method showed that all DENV-2 strains used in this study belong to cosmopolitan genotype A (genotype IV A) and are closely related to the strains circulating in the Philippines in 2001 (S1 Fig). Interestingly, we found that a group of viruses isolated in 2001 clustered with those from 2002 (i.e., TW/915, TW/1030), whereas the rest of the 2001 DENV-2 viruses formed an independent clade. Regardless if the sequence was from E or ORF genes, time-scaled Bayesian MCC analysis generated similar results with high posterior probabilities from both data sets (Fig 2A and 2B, respectively). These clusters were defined as groups Ia, Ib and II. The consensus sequence of these three groups showed genetic markers across the complete ORF (Table 1). Viruses classified as group Ib maintained a 2001-like amino acid signature at position E-46: threonine at position 46, compared to isoleucine in group II viruses; additionally, the Ib viruses also had two 2002-like signatures in NS5 protein (Table 1).

**Table 1:** Specific variants among the three groups of DENV-2 isolates from southern Taiwan, 2001 to 2003.

Genome		2001		2002		Amino acid	
		Clade I			Clade II		
		Ia	Ib		II		
position	Gene	consensus sequence				Position	Change
**1073**	**E**	**C**	**-**	**-**	**T**	**46**	**T→I**
1227	E	T	A	A	A	97	No change
2064	E	T	T	C	C	376	No change
3204	NS1	T	-	-	C	261	No change
6561	NS4A	C	-	-	T	62	No change
6912	NS4B	C	-	-	T	29	No change
7491	NS4B	T	-	-	C	222	No change
8349	NS5	C	T	T	T	260	No change
**8381**	**NS5**	**T**	**C**	**C**	**C**	**271**	**I→T**
**8639**	**NS5**	^**a**^**T/A**	**A**	**A**	**A**	**357**	**V→E**
8781	NS5	G	A	A	A	404	No change
9186	NS5	C	-	T	T	539	No change

Nucleotide changes among three groups of DENV-2 isolates collected at different time points from September 2001 to March 2003, in Kaohsiung/Pingtung, Taiwan. Three non-synonymous mutations were identified: E-46, NS5-271, and NS5-375.

^a^Four or three of seven clade Ia viruses had T or A at nt-8639 (NS5-357) position, respectively.

### Temporal and spatial monitoring of viral quasi-species changes among three groups of DENV-2 isolates in Kaohsiung/Pingtung from 2001 to 2003

**Fig 3:**
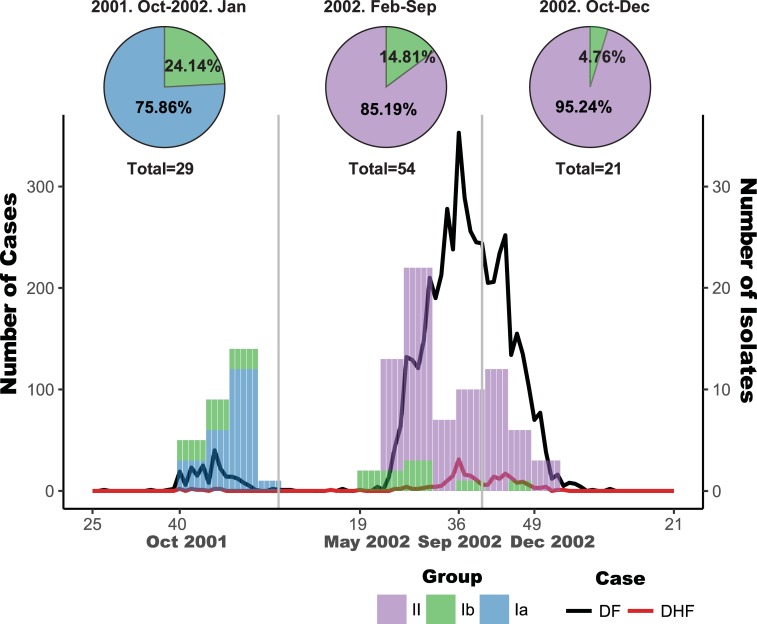
Temporal distributions of the three groups of DENV-2 viruses. Dynamic changes of the three groups of DENV-2 variants isolated from patients over time. At the peak of 2001 outbreak, the group Ia and Ib variants accounted for about 75.86% and 24.14% of the isolates, respectively. The earliest Ib variant was identified in November 2001, whereas the first confirmed dengue case occurred in July 2001. The group Ib variants remained circulating after 2001 outbreak, whereas the group II variants became dominant viruses during the outbreak period from February 2002 to September 2002, accounting for more than 85.19% of the isolates. After October 2002, 95.24% of the isolates belonged to group II.

To further understand the dynamic change of Ia, Ib and II groups of viruses, we analyzed each viral group based on the onset dates and residential locations of the dengue patients. Group Ib co-circulated with the other two groups throughout the 2001–2003 epidemic. At the peak of the 2001 outbreak, group Ia and Ib viruses accounted for 75% and 25% of the isolates, respectively (Fig 3). Group II represented the dominant viruses during 2002 to 2003, while group Ib viruses remained circulating toward the end of 2002 and accounted for only 5–15% of the isolates among 75 available viruses sequenced (Fig 3).

**Fig 4:**
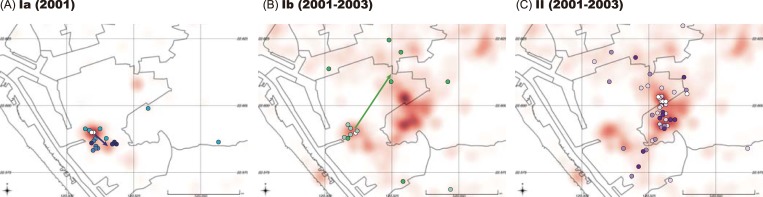
Differences in the pattern of geographical distribution of group Ia, Ib and II variants. Dots on the map indicate locations of patients’ residential areas. The intensity of color for each dot represents the chronology for the three groups of viral variants during their circulating period (A-C). The arrow on the map indicates the direction of virus spread based on the locations of MRCAs inferred in the MCC trees (A and B). Compared with groups Ia and Ib, the group II viral variants do not show a simple linear spreading route but did spread from the center of the clusters. Total isolates were plotted as dots in the indicated period, in colors blue, green and purple representing the groups Ia, Ib and II, respectively. The red shaded area illustrates all laboratory-confirmed dengue cases in the respective periods; the darker shading reflects higher case numbers.

We applied the continuous diffusion model to investigate the geographical dispersion of the three virus groups using the phylogeographic analysis program implemented in BEAST. The group Ia viruses clustered with the group Ib variants during the early stage of the epidemic but moved slightly toward the southeast in 2001 (Fig 4A), whereas the group Ib viruses expanded north-eastward after 2001 (Fig 4B). The group II viruses first emerged to the east of the epicenter, clustered with Ia and Ib virus variants, and then diffused irregularly outward from the center. The phylogeographic analysis also revealed that group Ia and II variants were the dominant virus populations in the 2001 and 2002–2003 outbreaks, respectively. Additionally, group Ib viruses were persistently present as a minor population in both outbreaks.

### Phylodynamic changes of the three groups of DENV-2 variants through the entire epidemic process

**Fig 5:**
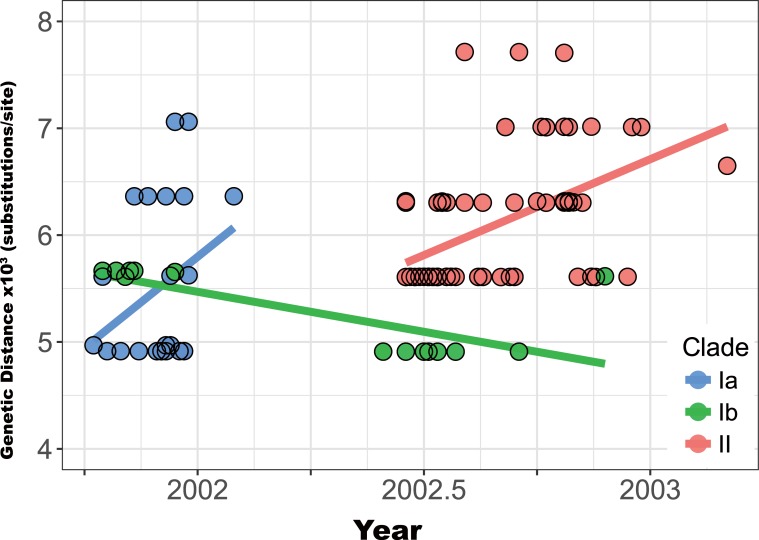
Genetic distances by isolation date among three groups of DENV-2 viral variants. Genetic distance, based on the E gene of each group of viral variants, was analyzed by linear regression. Solid lines show the estimated regression intervals. Group Ib variants had lower evolution rates (substitutions/site/year), with fewer genetic changes, compared with those of group Ia and II variants. The difference in estimated rates between the E protein and the ORF (S2 Fig) of Ia variants may be due to insufficient sample size and the fact that most genetic variations of group Ia viruses were found in the E gene.

Since the three groups of viruses had different spatio-temporal population dynamics, we applied phylodynamic analysis to compare their genetic distances, substitution rates, and transmission capabilities (R). When genetic distances for the E gene relative to the inferred common ancestor were plotted against time, the persistent Ib variants showed a lower rate of accumulating substitutions through time (with decreasing trend in the genetic distance) compared to those of groups Ia and II variants (Fig 5 and S2 Fig). The mean evolutionary rates in the E gene of group Ib, calculated by Bayesian analyses, were also consistently lower than those of the other two variant groups (Ib: 1.06E-3; Ia: 2.76E-3; II: 2.27E-3; Table 2), with no statistically significant difference.

**Table 2:** Evolutionary characteristics of the E gene of the three DENV-2 variants.

Group of DENV-2 Variants	Divergence according to the indicated evolutionary model^a^	
	Constant size (TN93+G4)	
	Substitution rate	95% HPD Interval^b^
Ia	2.76E-03	[3.8001E-8, 8.15E-3]
Ib	1.06E-03	[3.6998E-5, 2.5118E-3]
II	2.27E-03	[9.4835E-4, 3.7959E-3]

^**a**^ Based on strict coalescent inference. See Materials and Methods and S2 Table for more details of the estimation procedure.

^**b**^ 95% Highest Posterior Density (HPD) Interval.

**Fig 6:**
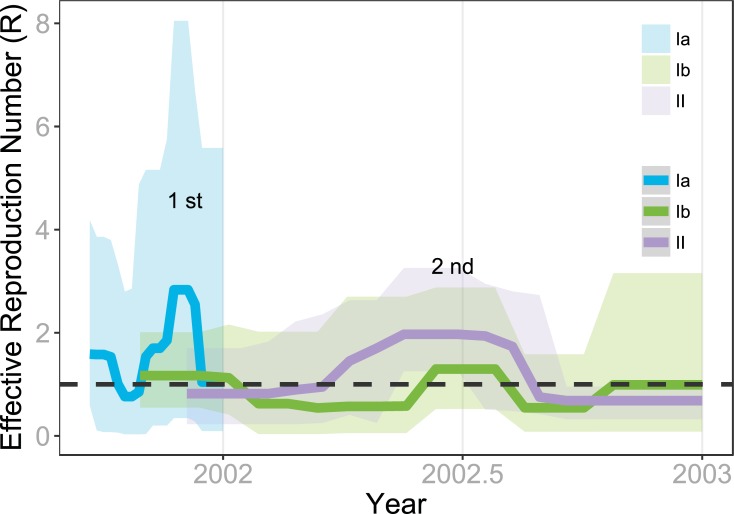
Viral transmissibility of the three groups of DENV-2 variants measured by effective reproductive number (R). The R (solid line) values and 95% HPD interval (shaded area) from 2001 to 2003 of three DENV-2 viral variants were estimated by birth-death skyline serial analysis. The plot clearly demonstrates that the two peaks of viral transmissibility corresponded well with the two peaks of confirmed DENV-2 cases from 2001 to 2003. The R values of both group Ia and Ib variants surpassed 1.00 during 2001. They were replaced by group II variants when R surged in early 2002, accompanied by the recurrence of group Ib variants.

The effective reproduction number (R) provides an important epidemiological parameter to measure the viral transmissibility. The birth-death skyline serial model was implemented to estimate R for the three groups of viral variants. The estimated R values corresponded well to the number of confirmed dengue cases during the entire epidemic period (Fig 6). Both R values of group Ia and Ib viral variants surpassed 1.00 during the first outbreak in 2001 and decreased to below 1.00 after 2001. Only group Ib’s R value rose above 1.00 in mid-2002 when the case number reached a peak in Fig 6 and the group II variants (R >1) replaced the group Ia as the dominant variant during 2002. Notably, low transmissibility of the Ib variant after 2001 partially explained its lack of isolation from dengue patients. Therefore, the three independent measures (lower genetic distance, substitution rate, and transmissibility) show clearly that group Ib viral variants not only co-circulated with Ia and II viral variants but also continuously persisted throughout these two separate DENV-2 outbreaks from 2001 to 2003.

### Temporal patterns identified dynamics of intra-host viral sub-populations using deep sequencing

How different genetic variants (group Ia, Ib, and II) emerged through transmission was further investigated within human hosts using deep sequencing. The E gene was selected as the target gene to study intra-host viral populations for the following reasons. First, E gene nucleotide sequences (Fig 2A) exhibited a similar topology of the phylogenetic tree as using complete ORF (Fig 2B) in our phylogenetic analysis. Second, there are three specific markers in E protein that can be used to distinguish different clades instead of NS5 or other genes (Table 1). Third, previous publications suggested that the E gene has the higher sequence heterogeneity among genes of the dengue genome [37–40]. Fourth, the E protein on the outer virion surface plays an important role for virus entry and eliciting protective immunities after infection.

**Fig 7:**
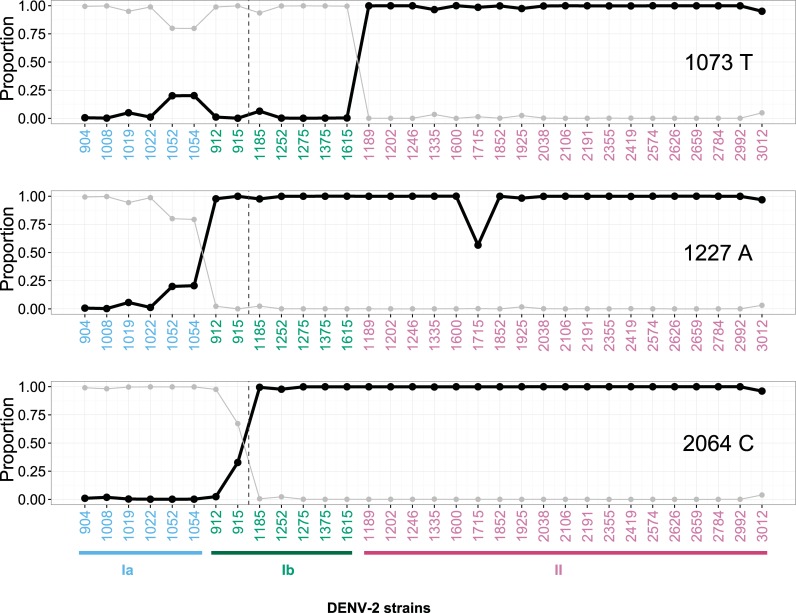
Deep sequencing of evolving viral populations. Proportions of quasi-species variants of each virus isolate (x-axis) are illustrated by three nucleotide positions in E-1073, -1227 and -2064. Proportions of Thymine (T) at position 1073 (upper), Adenine (A) at position 1227 (middle) and Cytosine (C) at position 2064 (lower) are shown separately in three panels, respectively, by black dots. Their corresponding putative nucleotide substitution counterparts - 1073C, 1227T, and 2064T are shown in gray dots. Three phylogenetic groups of DENV-2 variants are highlighted by different colors (Ia-blue, Ib-green, and II-purple) on the x-axis. Viruses are arranged by the chronological order of their isolation dates within each group. The vertical dashed line separates viruses isolated in 2001 (left side) versus those in 2002 (right side).

Fig 7 shows the proportions of nucleotide variants obtained by deep sequencing among three variant groups during two consecutive outbreaks. The proportion of the C1073T substitution was increased in the group Ia quasi-species sequence during the first 2001 outbreak (5.90%, 20.5% and 21.1% of C1073T were found in strains-1019, -1052 and -1054, respectively). The C1073T substitution was also detected in the group Ib virus variants in 2002 (strain-1185; 7.29%). For the T1227A substitution (where Ib and II shared a 1227A signature), the proportion of 1227A substitution in the Ia strains-1019, -1052 and -1054 increased from 6.87% to 22.3%. A similar temporal pattern was also observed at the T2064C substitution for the group II-variants, from 32.3% of strain-915 to 100% of all other 2002 group II-like viruses.

**Fig 8:**
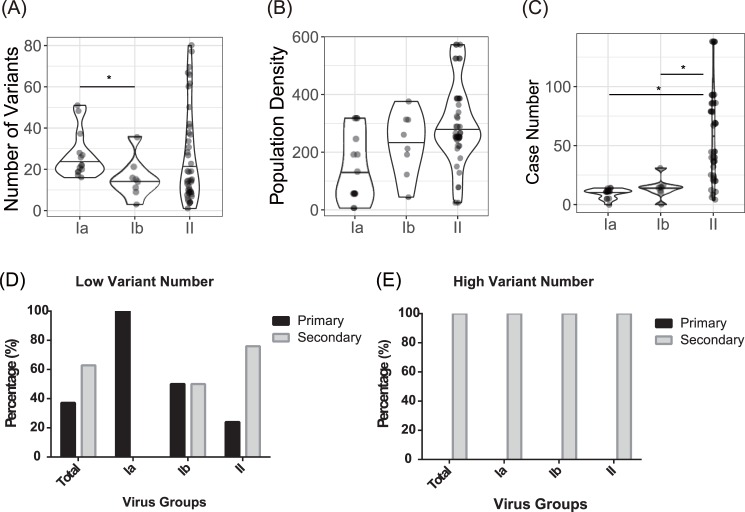
Factors associated with viral diversity: Population sizes, associated cases and population densities and secondary infection. (A) Distributions in the numbers of three groups of DENV-2 variants. The number of variants in group Ib was significantly lower than in group Ia viral variants. (B) Distribution of Li-specific population densities. (C) Li-specific numbers of dengue cases. Numbers of dengue cases associated with the group Ia and Ib variants were significantly lower than with group II. The Mann–Whitney–Wilcoxon test was used and significant differences (p<0.05) were labeled with an asterisk. The horizontal bar in the middle of the violin plots indicates the median values of the three groups. Proportions of primary and secondary DENV infections within each group of viruses classified according to the numbers of the two groups: (1) low variant (< 20) (D), and (2) high variant (≥20) (E), boundary by medium value (medium is 20). (E). Primary DENV infection accounted for percentages of dengue cases in the low variant group, particularly in Ia variants. However, secondary DENV infection increased its percentages in the low variant groups of Ib and II viruses and eventually accounted for 100% of the secondary infection group for all the Ia, Ib and II groups with a high variant number.

Previous studies suggested that the increase of quasi-species complexity is associated with population density and the presence of heterotypic antibodies elicited by secondary DENV infection in the affected population [40, 41]. Salje et al. proposed a method for transmission chains estimated by phylogenetic and serological data in Thailand. They proposed inter-host viral diversity increases with population density through transmission chains [41]. To further understand how viral variants emerged from infected human hosts and their relationship with transmission intensity, the population density and numbers of dengue cases from their residential districts, numbers of variants, and the patient’s past immunity to the infection (primary vs secondary DENV infection) were investigated. We analyzed 64 samples from three districts including Lingya, Qianzhen and Fengshan District, where a large number of dengue cases were concentrated in 2001–2002 epidemics. In addition, we used the official geographical division “Li” as the spatial unit to analyze potential correlations between spatial or epidemiological characteristics and quasi-species. Case number refers to annual patient numbers in the particular Li where the virus was isolated. The results showed that the group Ib variants had significantly lower numbers than Ia (p<0.05) (Fig 8A). Both group Ia and Ib had significantly lower cases numbers in residential “Li” compared with group II (p<0.05) (Fig 8C). There was no significant difference in population density of a particular “Li” between three variant groups (Fig 8B). However, Group II viruses had a wide spectrum of variant numbers, population density and case numbers (Fig 8A–8C), which possibly contributed to the pervasive dispersal pattern of the viruses (Fig 4). In addition, the proportion of variants in group II increased sharply, which correlated with striking increases in the case numbers (Fig 8C), indicating that transmission intensity might play an important role in increasing viral diversity as well as the magnitude of an epidemic. Although wide distributions of variant numbers from all three groups of viral variants were observed, the group with highest variant numbers (≥20) were exclusively isolated from the secondary DENV patients (Fig 8D and 8E).

## Discussion

Phylodynamic inferences based on viral sequence data with spatio-temporal dynamics can reveal the characteristics of viral transmission in a local urban setting [42]. Limited sample numbers have prevented previous studies from effectively determining the inter- and intra-host diversity and evolution of dengue virus during outbreaks [40, 43]. In this study, we applied the deep sequencing technique to obtain 104 viral E gene sequences amplified directly from acute patients’ serum samples and phylodynamic analysis identified three genetic variants (groups Ia, Ib and II). Although the clade replacement of group Ia by group II during the two consecutive outbreaks was consistent with previous findings [44], our deep sequencing results were the first to reveal the population dynamics associated with emergence and co-existence of different genetic variants within human hosts. Furthermore, the clade replacement and co-existence of different viral variants strongly implied that viruses contributing to the second peak of the 2002–2003 outbreak were likely to have evolved from viruses that circulated in 2001 in Kaohsiung, not from another introductive event.

Deep sequencing technologies capable of sequencing individual molecules directly from PCR amplicons shows unprecedented resolution for studying quasi-species within viral populations. Previous studies utilizing this new technology demonstrated that the purifying selection of dengue viral evolution with variants consistently showed higher intra-host genetic diversity than inter-host diversity [40, 45–47]. Three genetic variants (group Ia, Ib and II) identified by phylodynamic analysis revealed three amino acid changes (E-46, NS5-271 and, NS5-357). The bottleneck transmission resulted in mutation fixation and clade replacement has been observed in a previous DENV-3 study [48]. Furthermore, the transition to ≥20% of variants at these specific positions emphasizes that the epidemic process might have selected and amplified variant(s) with higher epidemic potential during this outbreak. It is very likely that epidemiological conditions such as high transmission intensity and high viral load might result in the fixation of the nonsynonymous substitution at the C1073T position in the E protein. This mutation is a significant genetic marker correlated with both clinical and epidemiological severities in this study and a previous study [44]. Therefore, that the combination of a consensus sequence to identify the major viral variant among quasi-species and deep sequencing to analyze the breadth of quasi-species variants may make it possible to monitor and identify emerging variants with higher epidemic potential prior to a larger-scale transmission or outbreak.

The process of clade replacement may be influenced by the host immune status (primary or secondary DENV infection) and the transmission intensity within communities [41, 48]. Under these circumstances, viral fitness changes during transmission of alternating host species at a population level or through disease progression at an individual level (S3 Fig). Intra-host infections with higher numbers of quasi-species variants could offer an advantage for arboviruses to adapt as they cycle between two very different host species. Detailed analysis of the spectrum of quasi-species variants within an individual patient’s serum found that groups containing high numbers of variants (≥20), regardless of a Ia, Ib, or II variant, exclusively emerged from the patients experiencing secondary DENV infection (Fig 8D and 8E) which is consistent with a previous study [48]. Guzman et al. proposed the role of the immune-escape mutant for contributing to the rapidly increasing fatality rates in Cuba in 2000 [49]. Supported by a previous study [50], we hypothesized that viruses carrying 46I could be less optimally neutralized than 46T viruses by cross-reactive, neutralizing antibodies elicited during prior DENV infections. This difference in cross-reactive, neutralizing antibodies may subsequently confer a selective advantage to the 46I virus in a community that experienced prior dengue infections. Our study similarly suggests that heterotypic antibodies drive the evolution of dengue viruses and increase a variant population that may lead to increased severity of the epidemic.

We confirmed the ancestral role of the group Ia variants as contributing to the subsequent larger epidemic beginning in 2002. Between the two peaks of the outbreaks (week 8 to 13 in 2002), there were no confirmed indigenous dengue cases. However, it is noteworthy mentioning that asymptomatic or pre-symptomatic dengue patients do retain their transmissibility to mosquitoes, and even have higher transmissibility to mosquitoes than symptomatic patients [51]. With such a high asymptomatic rate of dengue virus infection [5], it is likely that the overwintered Ib variants might cause asymptomatic infections and maintain silent transmission in the population. Transitional Ib virus variants exhibited several features possibly favoring overwintering: low transmissibility (lower R), low quasi-species complexity (lower genetic divergence since emergence), less potential to cause severe disease (S5 Table), and circulating in a non-cluster region (Figs 4B and 8C). Additionally, the Ib variant maintained a higher virus load compared to those of the Ia and II variants in acute-phase patients’ plasma, which might imply the stability and maintenance role of Ib viruses (S4 Fig). Thus, group Ib variants may play a key overwintering role by maintaining a population in a certain ecological niche in the urban setting during dengue epidemics.

This study has three major limitations. First, we focused on viral population dynamic changes within only human hosts. We were not able to obtain virus from mosquitoes due to the limitation of the mosquito surveillance system and the low isolation rate. Second, we only collected the plasma samples from dengue patients. We did not have plasma samples from pre-clinical or asymptomatically infected persons so that we could not investigate the virus dynamics in pre-clinical and asymptomatically patients’ specimens to address the role of silent transmission during the epidemic process. Third, we performed deep sequencing targeting only the E protein gene (rather than full-length viral genome) to increase sample sizes and to increase the quality of sequences obtained directly from patients’ plasma. Fourth, mosquito density may influence the intensity of dengue transmission. Unfortunately, due to a lack of a mosquito surveillance data, we are not able to conduct additional analysis.

Few amino acid substitutions have been shown to associate with increased epidemic scale and disease severity caused by several arboviruses, such as such as West Nile, dengue, chikungunya, and Zika [52–57]. Possible mechanisms may exist to maintain viral populations under stressful environmental conditions (e.g., winter season), then subsequently expand viral transmission to increase the population diversity, followed by selection of a viral variant with higher replication and/or transmissibility potential. Arboviruses capable of overcoming these barriers, such as a variant virus associated with greater pathogenesis and disease severity potential, could evolve under a combination of favorable epidemiological conditions [32, 58].

In this study, we identified three amino acid substitutions, T46I in the E protein, I271T, and V357E in the NS5 protein (Table 1). E-46 position is located in the D0 beta strand of domain I on the protein surface; domain I is the structurally central domain of E protein which plays an important role in protein stability [59, 60]. Threonine, a polar amino acid, was substituted by isoleucine which is non-polar but retains the property of having a chiral carbon in the side chain. This T46I change would stabilize the protein structure [61]. Furthermore, this residue is located in a T-helper cell epitope [50]. Mutation in this residue may affect neutralizing antibody binding to viruses and T-cell recognition by the human host. Additionally, the previous study indicated that genetic variations in human-derived viral quasispecies mainly occurred in structural proteins (prM, E, and NS1), whereas mosquito-derived variations occurred in NS3 and 3ʹ UTR [62]. We thus hyphothesize that the T46I mutation emerged under human immune pressure during epidemic transmission. Viruses with the point mutation may have potentially evaded immune responses and this may have resulted in higher virulence and transmissibility when compared to non-mutant viruses. There is only one amino acid difference (46 in E) between group Ib and group II virus-consensus sequences, and group Ib has lower transmissibility and lower numbers of DHF cases compared with group II viruses (S5 Table). Two other amino acid changes are located at positions 271 and 357 in the NS5 protein. The NS5 protein contains the N-terminal methyltransferase and the C-terminal RNA-dependent-RNA polymerase (RdRp) domains involved in viral replication [63]. Amino acid positions 271 and 357 are located in the inter-domain linker region (residues 264 to 273 in DENV-2) and inter-domain interface (residues 349 to 358 in DENV-2), respectively. These two inter-domain regions determine the flexibility of the NS5 protein essential for virus replication [64–66]. Additionally, V357E changes from nonpolar (valine) to negative charge amino acid (glutamate) are located in a conserved β nuclear localization signal (βNLS) motif of the RdRp domain. This segment not only plays a role in transporting NS5 into the nucleus but also interacts with the NS3 helicase [63, 67]. Taken together, these two substitutions in NS5 may influence viral replication in different hosts. How these three amino acid substitutions might influence the transmissibility and virulence of viruses will be addressed in a future study. Based on our results, we hypothesize that Ib (with two amino acid changes in NS5, 271T and 357E, compared with Ia) has better replication ability and spread to the west-north of the cluster region in 2001 (Fig 4). Additionally, Ib caused low case numbers of symptomatic infection (S5 Table) that was barely detected by the existing surveillance system. In the winter period, when the mosquito density and biting behavior decrease, better replication could help viruses maintain in mosquitos. With low transmission rates, the virus accumulated variants slowly in both intra-host (low intra-host variant numbers) and inter-host (low genetic diversity).

In conclusion, our study tracked viral dynamics associated with inter-host and intra-host genetic changes encompassed with spatio-temporal trends using phylodynamic analysis and deep sequencing. These observations have direct implications for the evolution of DENV as well as other arboviruses and underscore the value of such investigations to map the high-resolution of viral quasi-species dynamics.

## Supporting information

S1 Table Primers for DENV-2 ORF sequencing. (DOCX)

S2 Table DENV viral strain sequences analyzed in the current study. (DOCX)

S3 Table Log marginal likelihoods of various models by different methods. (DOCX)

S4 Table Primers used in dengue envelope protein deep sequencing. (DOCX)

S5 Table Patients infected by group Ia, Ib and II viruses showed differences in degree of illness for the severe form of dengue. (DOCX)

S6 Table Information and viral sequences used in this study for the 2001–2003 epidemics. (DOCX)

S7 Table Correlations between intra-host diversity, immune status of hosts and disease severity. (DOCX)

S1 Fig Phylogenetic analyses of DENV-2 viruses in Taiwan, 2001–2003. Maximum-likelihood tree with bootstrap values was constructed from the 73 E gene (1485 nt), using 63 DENV-2 viruses and 10 other serotypes of DENVs isolated in Taiwan and other countries. The DENV-2 viruses causing the 2001–2003 epidemic belonged to cosmopolitan genotype A and clustered with Philippine strains.
(DOCX)

S2 Fig Genetic distances vs. time of the DENV-2 viruses isolated during the 2001–2003 epidemics. We used linear regression to analyze the genetic distance of viruses belonging to groups Ia, Ib and II based on the Open Reading Frame (ORF) sequence of each virus. Solid lines show estimated regression intervals. Group Ib has a lower evolution rate (substitutions/site/year) for the E protein compared with groups Ia and II, e.g. fewer genetic changes were observed for the E protein during the study period (Fig 5). The difference in estimated evolution rates of Ia viruses using E or ORF sequences may be due to the small sample size or that the E gene encodes a higher number of variations than the ORF gene.
(DOCX)

S3 Fig Inter-host (population) and intra-host (individual) genetic diversity of the DENV-2 viruses isolated among Ia, Ib and II groups viruses. Inter-host viral genetic diversity was evaluated by modified pi, calculated by DnaSP v5 software package for the analysis of nucleotide polymorphism from aligned DNA sequence data. Intra-host viral genetic diversity was evaluated by modified pi. The three groups of viruses share different patterns (i.e. groups Ia and Ib viruses have lower genetic diversity than group II viruses) in both inter-host and intra-host diversity. Red dots indicate samples isolated in the acute phase (0–3 days after onset of illness) and blue dots indicate samples isolated in the defervescence phase (4 days after illness) with an intra-host diversity value of pi. Open diamonds indicate the inter-host diversity of the E region sequences of all three virus groups.
(DOCX)

S4 Fig Viral loads and viral population variants found in the three virus groups, Ia, Ib and II. (A) Viral load in the group II viruses was lower than the other two groups of viruses during the acute phase (0 to 3 days after onset of illness), viral load decreased significantly after the acute phase (defervescence phase, 4–8 days after onset of illness). (B) Group Ib had significantly lower variant numbers than group Ia during the acute phase. * indicated p<0.05. Statistical analysis (two-tailed student t-test) comparing the different virus groups was completed.
(DOCX)

S5 Fig Bayesian Skyride Plot and the number of confirmed dengue cases, from July 2001 to Jan. 2003. Bayesian Skyride plot analysis was employed and the effective population size (Ne.g) of DENV-2 is shown by a solid line with 95% highest posterior density (HPD) intervals (Shaded region) (Right axis). The bar plot indicates the number of confirmed dengue cases from 2001 to 2003 in southern Taiwan (Left axis). Effective population size based on the Bayesian method agrees well with Epi-curve analysis.
(DOCX)

S6 Fig Coverage and distribution of E gene variants identified by deep sequencing. (A) cDNA libraries were generated by four overlapping fragments, shown as dashed lines, and were used for deep sequencing. Blue and red squares indicate the location of forward primers and reverse primers, respectively. The solid black line indicates median with gray lines showing the first (Q1) (The lowest 25% of numbers) and third (Q3) (The 75% of numbers) quartiles. (B) Distribution of variants detected by LoFreq at each nucleotide position for all analyzed samples. (C) Comparisons of median coverage versus number of variants for all sequenced samples.
(DOCX)
