# Supplementary material for: Inter- and intra-host sequence diversity reveal the emergence of viral variants during an overwintering epidemic caused by dengue virus serotype 2 in southern Taiwan
Source: PLoS Negl Trop Dis. 2018 Oct 4;12(10):e0006827. doi: 10.1371/journal.pntd.0006827 (PMC6191158; doi:10.1371/journal.pntd.0006827)
Supplement: S1 Table — (DOCX) [file pntd.0006827.s001.docx]

**S1 Table. Primers for DENV-2 ORF Sequencing**

| **PCR fragment**  **Size (bp)** | **Primer** | **Primer sequence** |
| --- | --- | --- |
| FID1  809 bp | d2s1C^C^ | GAT GAG GGA AGA TGG GGA GTT GTT AGT CTA CGT GGA C |
|  | d2M27B^b^ | TTT CCA GGC CCC TTC TGA TGA CAT CCA |
| FID2  1984 bp | d2-518F | ACC CTT ATG GCC ATG GAC CTT G |
|  | d2E+34B^b^ | GGA ACT TGT ATT GTT CTG TCC |
| FID2 ^a^ | d2-E420F | GAT AAC ACC TCA CTC AG |
|  | d2-E712R | CCA ATG TCT CCT TCT GTA TC |
| FID3  1923 bp | d2-2000F | CAG CCC AGT CAA CAT AGA AG |
|  | d2-3921B^b^ | GGG ACG CAC AAG ATG GCC |
| FID4  1539 bp | d2-2940A^b^ | CAA AAC TCA TGT CCT AAT GC |
|  | d2-4512B^b^ | ACA GGT ACC ATG CTG CTG C |
| FID5  1682 bp | d2-4119A^b^ | ARC AAG AAA AGG AGC TGG CC |
|  | d2-5812B^b^ | CGT CTG GGG TCT ATA ACC C |
| FID6  1475 bp | d2-5425F | CAT GAC AGC TAC TCC TCC TG |
|  | d2-6911R | CTG ATG CAG GAC GTA GAT C |
| FID7  1866 bp | d2-5798F | CTA ACA GAC GGT GAA GAG CG |
|  | d2-7663R | GGT TCT ATC CAC CTC CTG |
| FID8  1644 bp | d2-7240A^b^ | GCG GGC ATC ATG AAA AAC CC |
|  | d2-8885B^b^ | TTC CTT GTC AAC CAG CTC CC |
| FID9  1785 bp | d2-8585A^b^ | ACA GAT GGC AAT GAC AGA CAC |
|  | d2-10370B^b^ | TAA CGT CCT TGG ACG GGG |
| FID10  500 bp | d2-10204A^b^ | TAC ACA GAT TAC ATG CCA TCC |
|  | d2-10704B^b^ | CAC CAT TCC ATT TTC TGG CG |

a. Ten primer pairs designed to amplify and sequence ORF of DENV-2 virus. FID2 fragment is the longest of 10 fragments. Two internal primers were used to improve the quality of sequence.

b. Primers from reference [22].

c. Primers from reference [S1 Table reference 1].

References

1. Christenbury JG, Aw PP, Ong SH, Schreiber MJ, Chow A, Gubler DJ, et al. A method for full genome sequencing of all four serotypes of the dengue virus. Journal of virological methods. 2010;169(1):202-6.
