# Supplementary material for: Inter- and intra-host sequence diversity reveal the emergence of viral variants during an overwintering epidemic caused by dengue virus serotype 2 in southern Taiwan
Source: PLoS Negl Trop Dis. 2018 Oct 4;12(10):e0006827. doi: 10.1371/journal.pntd.0006827 (PMC6191158; doi:10.1371/journal.pntd.0006827)
Supplement: S2 Table — (DOCX) [file pntd.0006827.s002.docx]

**S2 Table. DENV viral strain sequences analyzed in the current study**

| **Strain** | **Country** | **Isolation Year** | **Accession Number** | **Gene** |
| --- | --- | --- | --- | --- |
| D2/PG/New Guinea C/44 | Papua New Guinea | 1944 | AF038403.1 | E |
| D1/ID/A88/88 | Indonesia | 1988 | AB074761.1 | E |
| D2/IN/96-19-1HuNIID/96 | India | 1996 | AB111449.1 | E |
| D2/LK/00-43-1HuNIID/00 | Sri Lanka | 2000 | AB111452.1 | E |
| D2/ID/01-04-1HuNIID/01 | Indonesia | 2001 | AB111453.1 | E |
| D2/JM/77/2007NIID/07 | Jamaica | 2007 | AB545873.1 | E |
| D2/CK/Cook Islands 1/97 | Cook Islands | 1997 | AF004020.1 | E |
| D2/VE/Ven2/87 | Venezuela | 1987 | AF100465.1 | E |
| D2/MY/P8-1407/70 | Malaysia | 1970 | AF231717.1 | E |
| D2/GN/PM33974/81 | Guinea | 1981 | AF231719.1 | E |
| D2/SN/DAKHD10674/70 | Senegal | 1970 | AF231720.1 | E |
| D4MY/P75-514/75 | Malaysia | 1975 | AF231723.1 | E |
| D4/DM/Dominica 1981/81 | Dominica | 1981 | AF326573.1 | E |
| D2/VE/LARD1910/97 | Venezuela | 1997 | AF363081.1 | E |
| D2/VE/lard5942/00 | Venezuela | 2000 | AF398111.1 | E |
| D2/VN/CTD28/97 | Viet Nam | 1997 | AF410353.1 | E |
| D2/TW/768230/87 | Taiwan | 1987 | AF435481.1 | E |
| D2/PH/SLMC_148/95 | Philippines | 1995 | AY512569.1 | E |
| D2/PE/IQT2133/96 | Peru | 1996 | AY577439.1 | E |
| D2/PH/Phil03/03 | Philippines | 2003 | AY706007.1 | E |
| D2/PH/01RBD1/01 | Philippines | 2001 | AY786376.1 | E |
| D2/PH/01St321/01 | Philippines | 2001 | AY786377.1 | E |
| D2/PH/01St322/01 | Philippines | 2001 | AY786378.1 | E |
| D2/PH/01St428/01 | Philippines | 2001 | AY786394.1 | E |
| D2/PH/02Sa32/02 | Philippines | 2002 | AY786395.1 | E |
| D2/PH/00St68/00 | Philippines | 2000 | AY786403.1 | E |
| D2/PH/00U18/00 | Philippines | 2000 | AY786404.1 | E |
| D2/PH/NCH35/00 | Philippines | 2000 | AY786405.1 | E |
| D2/PH/NCH57/00 | Philippines | 2000 | AY786406.1 | E |
| D2/ID/BA05i/04 | Indonesia | 2004 | AY858035.2 | E |
| D2/ID/TB16i/04 | Indonesia | 2004 | AY858036.2 | E |
| D2/CN/Zhejiang/01/04 | China | 2004 | AY871813.1 | E |
| D4/PH/H241/56 | Philippines | 1956 | AY947539.1 | E |
| D2/TH/ThD2_0014/79 | Thailand | 1979 | DQ181809.1 | E |
| **Strain** | **Country** | **Isolation Year** | **Accession Number** | **Gene** |
| D2/TH/ThD2_0165/82 | Thailand | 1982 | DQ181820.1 | E |
| D2/TH/ThD2_3010/96 | Thailand | 1996 | DQ181866.1 | E |
| D2/TH/ThD2_K0062/00 | Thailand | 2000 | DQ181893.1 | E |
| D2/MX/3315/94 | Mexico | 1994 | DQ341196.1 | E |
| D2/MX/Borneo/620/88 | Mexico | 1988 | DQ341200.1 | E |
| D2/TW/806KH0110a/Tw/01 | Taiwan | 2001 | DQ518630.1 | E |
| D2/PH/Phil0506a/Tw/05 | Philippines | 2005 | DQ518631.1 | E |
| D2/PH/0109aTw/01 | Philippines | 2001 | DQ518632.1 | E |
| D2/PH/Phil0509a/Tw/05 | Philippines | 2005 | DQ518633.1 | E |
| D2/VN/0301aTw/03 | Viet Nam | 2003 | DQ518641.1 | E |
| D2/TW/247TP9810a/98 | Taiwan | 1998 | DQ518644.1 | E |
| D2/KH/0307aTw/03 | Cambodia | 2003 | DQ518649.1 | E |
| D2/MM/0207aTw/02 | Myanmar | 2002 | DQ518651.1 | E |
| D2/BF/DAK_Ar_2039/80 | Burkina Faso | 1980 | EF105382.1 | E |
| D2/CI/DAK_Ar_A1247/80 | Cote d'Ivoire | 1980 | EF105383.1 | E |
| D2/NG/IBH11208/66 | Nigeria | 1966 | EF105387.1 | E |
| D2/TW/PL046/81 | Taiwan | 1981 | EF540856.1 | E |
| D2/PR/1328/77 | Puerto Rico | 1977 | EU056812.1 | E |
| D2/TH/01A00203/01 | Thailand | 2001 | EU117333.1 | E |
| D2/TH/01A00134/01 | Thailand | 2001 | EU117352.1 | E |
| D2/PH/0308aTw/03 | Philippines | 2003 | EU448418.1 | E |
| D2/PH/0710bTw/07 | Philippines | 2007 | EU448426.1 | E |
| D2/CN/GD01/03/03 | China | 2003 | FJ196853.1 | E |
| D2/SG/SG(EHI)DES20105/05 | Singapore | 2005 | GQ357783.1 | E |
| D2/TO/Tonga_1974/74 | Tonga | 1974 | HM582117.1 | E |
| D2/BR/BR39145/RJ/90 | Brazil | 1990 | HQ012538.1 | E |
| D2/TW/BID-V5056/08 | Taiwan | 2008 | HQ891024.1 | E |
| D2/PH/0910bTw/09 | Philippines | 2009 | JF967996.1 | E |
| D2/ID/Bali_2010/10 | Indonesia | 2010 | JN568243.1 | E |
| D2/TP/East_Timor_2002/02 | East Timor | 2002 | JN568255.1 | E |
| D2/PG/PNG_2003/03 | Papua New Guinea | 2003 | JN568266.1 | E |
| D2/AU/Torres Strait 2003/03 | Australia | 2003 | JN568278.1 | E |
| D2/VN/Vietnam_2010a/10 | Viet Nam | 2010 | JN568281.1 | E |
| D2/TW/807KH1008a/10 | Taiwan | 2010 | JQ403524.1 | E |
| D2/PE/OBS9638/PER/00 | Peru | 2000 | JX051766.1 | E |
| **Strain** | **Country** | **Isolation Year** | **Accession Number** | **Gene** |
| D2/PE/IQD2227/PER/02 | Peru | 2002 | JX051773.1 | E |
| D2/PH/01-St-206/01 | Philippines | 2001 | KF744397.1 | ORF |
| D1/HI/Hawaii/44 | Hawaii | 1944 | KM204119.1 | E |
| D3/PH/H87/56 | Philippines | 1956 | KU050695.1 | E |
| D2/TH/16681/84 | Thailand | 1984 | KU725663.1 | E |
| D3/PR/1340/77 | Puerto Rico | 1977 | L11434.1 | E |
| D3/LK/260698/89 | Sri Lanka | 1989 | L11437.1 | E |
| D3/TH/MK-315/87 | Thailand | 1987 | L11442.1 | E |
| D2/LK/SL1050/69 | Sri Lanka | 1969 | M24451.1 | E |
| D2/TW/806KH0110b/01 | Taiwan | 2001 | **MG457038** | E |
| D2/TW/812KH0110a/01 | Taiwan | 2001 | **MG457039** | E |
| D2/TW/830KH0110a/01 | Taiwan | 2001 | **MG457040** | E |
| D2/TW/806KH0110d/01 | Taiwan | 2001 | **MG457041** | E |
| D2/TW/802KH0111a/01 | Taiwan | 2001 | **MG457042** | E |
| D2/TW/812KH0111a/01 | Taiwan | 2001 | **MG457043** | E |
| D2/TW/806KH0111a/01 | Taiwan | 2001 | **MG457044** | E |
| D2/TW/830KH0111a/01 | Taiwan | 2001 | **MG457045** | E |
| D2/TW/830KH0112a/01 | Taiwan | 2001 | **MG457046** | E |
| D2/TW/806KH0112a/01 | Taiwan | 2001 | **MG457047** | E |
| D2/TW/812KH0206a/02 | Taiwan | 2002 | **MG457048** | E |
| D2/TW/840KH0206a/02 | Taiwan | 2002 | **MG457049** | E |
| D2/TW/812KH0207a/02 | Taiwan | 2002 | **MG457050** | E |
| D2/TW/840KH0207a/02 | Taiwan | 2002 | **MG457051** | E |
| D2/TW/812KH0207b/02 | Taiwan | 2002 | **MG457052** | E |
| D2/TW/807KH0207a/02 | Taiwan | 2002 | **MG457053** | E |
| D2/TW/806KH0209a/02 | Taiwan | 2002 | **MG457054** | E |
| D2/TW/812KH0209a/02 | Taiwan | 2002 | **MG457055** | E |
| D2/TW/806KH0201a/02 | Taiwan | 2002 | **MG457056** | E |
| D2/TW/806KH0205a/02 | Taiwan | 2002 | **MG457057** | E |
| D2/TW/830KH0205a/02 | Taiwan | 2002 | **MG457058** | E |
| D2/TW/802KH0206a/02 | Taiwan | 2002 | **MG457059** | E |
| D2/TW/830KH0207a/02 | Taiwan | 2002 | **MG457060** | E |
| D2/TW/802KH0208a/02 | Taiwan | 2002 | **MG457061** | E |
| D2/TW/813KH0209a/02 | Taiwan | 2002 | **MG457062** | E |
| D2/TW/806KH0209b/02 | Taiwan | 2002 | **MG457063** | E |
| D2/TW/813KH0210a/02 | Taiwan | 2002 | **MG457064** | E |
| **Strain** | **Country** | **Isolation Year** | **Accession Number** | **Gene** |
| D2/TW/830KH0210a/02 | Taiwan | 2002 | **MG457065** | E |
| D2/TW/814KH0211a/02 | Taiwan | 2002 | **MG457066** | E |
| D2/TW/811KH0211a/02 | Taiwan | 2002 | **MG457067** | E |
| D2/ID/0209aTw/02 | Indonesia | 2002 | **MG457068** | E |
| D2/TW/830KH0303a/03 | Taiwan | 2003 | **MG457069** | E |
| D2/PH/0308bTw/03 | Philippines | 2003 | **MG457070** | E |
| D2/TW/2130/02^#^ | Taiwan | 2002 | **MG599558** | E |
| D2/TW/1721/02^#^ | Taiwan | 2002 | **MG599559** | E |
| D2/TW/1715/02^#^ | Taiwan | 2002 | **MG599560** | E |
| D2/TW/1600/02^#^ | Taiwan | 2002 | **MG599561** | E |
| D2/TW/1464/02^#^ | Taiwan | 2002 | **MG599562** | E |
| D2/TW/1439/02^#^ | Taiwan | 2002 | **MG599563** | E |
| D2/TW/1409/02^#^ | Taiwan | 2002 | **MG599564** | E |
| D2/TW/1372/02^#^ | Taiwan | 2002 | **MG599565** | E |
| D2/TW/1364-2/02^#^ | Taiwan | 2002 | **MG599566** | E |
| D2/TW/1315/02^#^ | Taiwan | 2002 | **MG599567** | E |
| D2/TW/1299/02^#^ | Taiwan | 2002 | **MG599568** | E |
| D2/TW/1272/02^#^ | Taiwan | 2002 | **MG599569** | E |
| D2/TW/1246/02^#^ | Taiwan | 2002 | **MG599570** | E |
| D2/TW/1203/02^#^ | Taiwan | 2002 | **MG599571** | E |
| D2/TW/1019/01^#^ | Taiwan | 2001 | **MG599572** | E |
| D2/TW/3011/02^#^ | Taiwan | 2002 | **MG599573** | E |
| D2/TW/2691/02^#^ | Taiwan | 2002 | **MG599574** | E |
| D2/TW/2626/02 | Taiwan | 2002 | **MG599575** | E |
| D2/TW/2592/02^#^ | Taiwan | 2002 | **MG599576** | E |
| D2/TW/2584/02^#^ | Taiwan | 2002 | **MG599577** | E |
| D2/TW/2574/02^#^ | Taiwan | 2002 | **MG599578** | E |
| D2/TW/2534/02^#^ | Taiwan | 2002 | **MG599579** | E |
| D2/TW/2533/02^#^ | Taiwan | 2002 | **MG599580** | E |
| D2/TW/2419/02^#^ | Taiwan | 2002 | **MG599581** | E |
| D2/TW/2237/02^#^ | Taiwan | 2002 | **MG599582** | E |
| D2/TW/1461/02^#^ | Taiwan | 2002 | **MG599583** | E |
| D2/TW/1022/01^#^ | Taiwan | 2001 | **MG599584** | E |
| D2/TW/1066/01^#^ | Taiwan | 2001 | **MG599585** | E |
| D2/TW/1060/01^#^ | Taiwan | 2001 | **MG599586** | E |
| D2/TW/1054/01^#^ | Taiwan | 2001 | **MG599587** | E |
| **Strain** | **Country** | **Isolation Year** | **Accession Number** | **Gene** |
| D2/TW/1052/01^#^ | Taiwan | 2001 | **MG599588** | E |
| D2/TW/1049/01^#^ | Taiwan | 2001 | **MG599589** | E |
| D2/TW/1039/01^#^ | Taiwan | 2001 | **MG599590** | E |
| D2/TW/1025/01^#^ | Taiwan | 2001 | **MG599591** | E |
| D2/TW/904/01^#^ | Taiwan | 2001 | **MG599592** | ORF |
| D2/TW/915/01^#^ | Taiwan | 2001 | **MG599593** | ORF |
| D2/TW/921/01 | Taiwan | 2001 | **MG599594** | ORF |
| D2/TW/950/01^#^ | Taiwan | 2001 | **MG599595** | ORF |
| D2/TW/1008/01^#^ | Taiwan | 2001 | **MG599596** | ORF |
| D2/TW/1018/01^#^ | Taiwan | 2001 | **MG599597** | ORF |
| D2/TW/1024/01^#^ | Taiwan | 2001 | **MG599598** | ORF |
| D2/TW/1029/01^#^ | Taiwan | 2001 | **MG599599** | ORF |
| D2/TW/1030/01^#^ | Taiwan | 2001 | **MG599600** | ORF |
| D2/TW/1034/01^#^ | Taiwan | 2001 | **MG599601** | ORF |
| D2/TW/1202/02^#^ | Taiwan | 2002 | **MG599602** | ORF |
| D2/TW/1252/02^#^ | Taiwan | 2002 | **MG599603** | ORF |
| D2/TW/1180/02^#^ | Taiwan | 2002 | **MG599604** | ORF |
| D2/TW/1183/02^#^ | Taiwan | 2002 | **MG599605** | ORF |
| D2/TW/1185/02^#^ | Taiwan | 2002 | **MG599606** | ORF |
| D2/TW/1189/02^#^ | Taiwan | 2002 | **MG599607** | ORF |
| D2/TW/1222/02^#^ | Taiwan | 2002 | **MG599608** | ORF |
| D2/TW/1231/02^#^ | Taiwan | 2002 | **MG599609** | ORF |
| D2/TW/1275/02^#^ | Taiwan | 2002 | **MG599610** | ORF |
| D2/TW/1280/02^#^ | Taiwan | 2002 | **MG599611** | ORF |
| D2/TW/1335/02^#^ | Taiwan | 2002 | **MG599612** | ORF |
| D2/TW/1355/02^#^ | Taiwan | 2002 | **MG599613** | ORF |
| D2/TW/1375/02^#^ | Taiwan | 2002 | **MG599614** | ORF |
| D2/TW/1364-1/02^#^ | Taiwan | 2002 | **MG599615** | ORF |
| D2/TW/1421/02^#^ | Taiwan | 2002 | **MG599616** | ORF |
| D2/TW/1615/02^#^ | Taiwan | 2002 | **MG599617** | ORF |
| D2/TW/1905/02^#^ | Taiwan | 2002 | **MG599618** | ORF |
| D2/TW/1925/02^#^ | Taiwan | 2002 | **MG599619** | ORF |
| D2/TW/1945/02^#^ | Taiwan | 2002 | **MG599620** | ORF |
| D2/TW/1949/02^#^ | Taiwan | 2002 | **MG599621** | ORF |
| D2/TW/2106/02^#^ | Taiwan | 2002 | **MG599622** | ORF |
| D2/TW/2132/02^#^ | Taiwan | 2002 | **MG599623** | ORF |
| **Strain** | **Country** | **Isolation Year** | **Accession Number** | **Gene** |
| D2/TW/2191/02^#^ | Taiwan | 2002 | **MG599624** | ORF |
| D2/TW/2208/02^#^ | Taiwan | 2002 | **MG599625** | ORF |
| D2/TW/2457/02^#^ | Taiwan | 2002 | **MG599626** | ORF |
| D2/TW/2559/02^#^ | Taiwan | 2002 | **MG599627** | ORF |
| D2/TW/2566/02 | Taiwan | 2002 | **MG599628** | ORF |
| D2/TW/2587/02^#^ | Taiwan | 2002 | **MG599629** | ORF |
| D2/TW/2659/02^#^ | Taiwan | 2002 | **MG599630** | ORF |
| D2/TW/2747/02^#^ | Taiwan | 2002 | **MG599631** | ORF |
| D2/TW/2784/02^#^ | Taiwan | 2002 | **MG599632** | ORF |
| D2/TW/2992/02^#^ | Taiwan | 2002 | **MG599633** | ORF |
| D2/TW/3012/02^#^ | Taiwan | 2002 | **MG599634** | ORF |

*Bold phase indicated sequences obtained from the current study

^#^ Viral cDNAs directly amplified from viral RNAs extracted from patient plasma to perform deep sequencing of envelope gene
