# Supplementary material for: Inter- and intra-host sequence diversity reveal the emergence of viral variants during an overwintering epidemic caused by dengue virus serotype 2 in southern Taiwan
Source: PLoS Negl Trop Dis. 2018 Oct 4;12(10):e0006827. doi: 10.1371/journal.pntd.0006827 (PMC6191158; doi:10.1371/journal.pntd.0006827)
Supplement: S3 Table — (DOCX) [file pntd.0006827.s003.docx]

**S3 Table. Log marginal likelihoods of various models by different methods**

| **Clock Model** | **Coalescent Model** | **AICM** | **Harmonic Mean Estimator** | **Log Marginal Likelihood** | |
| --- | --- | --- | --- | --- | --- |
|  |  |  | **ln P(data \| model)** | **PS** | **SS** |
| Strict | constant | 8801.40 | -4367.02 | -4626.23 | -4628.42 |
|  | exponential | 8799.69 | -4367.78 | -4622.65 | -4624.27 |
|  | GMRF | 8985.03 | -4391.73 | -4731.19 | -4734.97 |
| Relaxed Lognormal | constant | 11659.27 | -5779.32 | -6021.03 | -6038.73 |
|  | exponential | 11636.30 | -5778.94 | -5980.23 | -6036.25 |
|  | GMRF | 11739.92 | -5779.97 | -6118.38 | -6116.91 |
| Relaxed Exponential | constant | 11677.41 | -5779.31 | -6043.91 | -6047.49 |
|  | exponential | 11636.30 | -5774.47 | -6040.19 | -6043.58 |
|  | GMRF | 11727.70 | -5787.56 | -6106.49 | -6103.33 |

Log marginal likelihood estimates different molecular clock and coalescent model combinations. Model comparison using Akaike information criterion-Monte Carlo (AICM) method [1], harmonic mean estimator method [2, 3], log marginal likelihood path sampling model selection (PS) and stepping-stone model selection (SS) [1, 4]. Clock model used strict clock model; coalescent models used were the parametric constant and Gaussian Markov random field (GMRF) skyride model in this study [5]. The comparison results shows strict clock model is more fitting then relaxed lognormal clock model and relaxed exponential clock model, strict and exponential coalescent models share the similar result in four comparison results.

References

1. Baele G, Lemey P, Bedford T, Rambaut A, Suchard MA, Alekseyenko AV. Improving the accuracy of demographic and molecular clock model comparison while accommodating phylogenetic uncertainty. Molecular biology and evolution. 2012;29(9):2157-67.

2. Newton MA, Raftery AE. Approximate Bayesian inference with the weighted likelihood bootstrap. Journal of the Royal Statistical Society Series B (Methodological). 1994:3-48.

3. Suchard MA, Weiss RE, Sinsheimer JS. Bayesian selection of continuous-time Markov chain evolutionary models. Molecular biology and evolution. 2001;18(6):1001-13.

4. Baele G, Li WLS, Drummond AJ, Suchard MA, Lemey P. Accurate model selection of relaxed molecular clocks in Bayesian phylogenetics. Molecular biology and evolution. 2013;30(2):239-43.

5. Minin VN, Bloomquist EW, Suchard MA. Smooth skyride through a rough skyline: Bayesian coalescent-based inference of population dynamics. Molecular biology and evolution. 2008;25(7):1459-71.
