# Supplementary material for: Inter- and intra-host sequence diversity reveal the emergence of viral variants during an overwintering epidemic caused by dengue virus serotype 2 in southern Taiwan
Source: PLoS Negl Trop Dis. 2018 Oct 4;12(10):e0006827. doi: 10.1371/journal.pntd.0006827 (PMC6191158; doi:10.1371/journal.pntd.0006827)
Supplement: S4 Table — (DOCX) [file pntd.0006827.s004.docx]

**S4 Table. Primers used in dengue envelope protein deep sequencing**

| **Primers** | **Fragment size (bp)** | **Position** | **Primer sequence** |
| --- | --- | --- | --- |
| Forward primer's Tag | | | TCGTCGGCAGCGTCAGATGTGTATAAGAGACAG |
| Revers primer's Tag | | | GTCTCGTGGGCTCGGAGATGTGTATAAGAGACAG |
| Tag-E1-F | 493 bp | 884 | TCG TCG GCA GCG TCA GAT GTG TAT AAG AGA CAG TCC CGA GAG TCC TGA TTT |
| Tag-E1-R |  | 1376 | GTC TCG TGG GCT CGG AGA TGT GTA TAA GAG ACA GTC CCC TGA GTG AGG TGT TAT |
| Tag-E2-F | 461 bp | 1310 | TCG TCG GCA GCG TCA GAT GTG TAT AAG AGA CAG TGG AAG GTA AAG TCG TGC |
| Tag-E2-R |  | 1770 | GTC TCG TGG GCT CGG AGA TGT GTA TAA GAG ACA GCA GTA AGT TTC CTG ATG AC |
| nTag-E3-F | 438 bp | 1616 | TCG TCG GCA GCG TCA GAT GTG TAT AAG AGA CAG AAG GAT CAA ATT GGA TAC AG |
| nTag-E3-R |  | 2053 | GTC TCG TGG GCT CGG AGA TGT GTA TAA GAG ACA GAT GGA GGT TCT GCT TCT AT |
| Tag-E4-F | 499 bp | 2026 | TCG TCG GCA GCG TCA GAT GTG TAT AAG AGA CAG CCA GTC ACC ATA GAA GCA G |
| Tag-E4-R |  | 2524 | GTC TCG TGG GCT CG GAG ATG TGT ATA AGA GAC AGG GAA CTT GTA TTG TTC TGT CC |
