# Supplementary material for: Inter- and intra-host sequence diversity reveal the emergence of viral variants during an overwintering epidemic caused by dengue virus serotype 2 in southern Taiwan
Source: PLoS Negl Trop Dis. 2018 Oct 4;12(10):e0006827. doi: 10.1371/journal.pntd.0006827 (PMC6191158; doi:10.1371/journal.pntd.0006827)
Supplement: S5 Table — (DOCX) [file pntd.0006827.s005.docx]

**S5 Table. Patients infected by group Ia, Ib and II viruses showed differences in degree of illness for the severe form of dengue.**


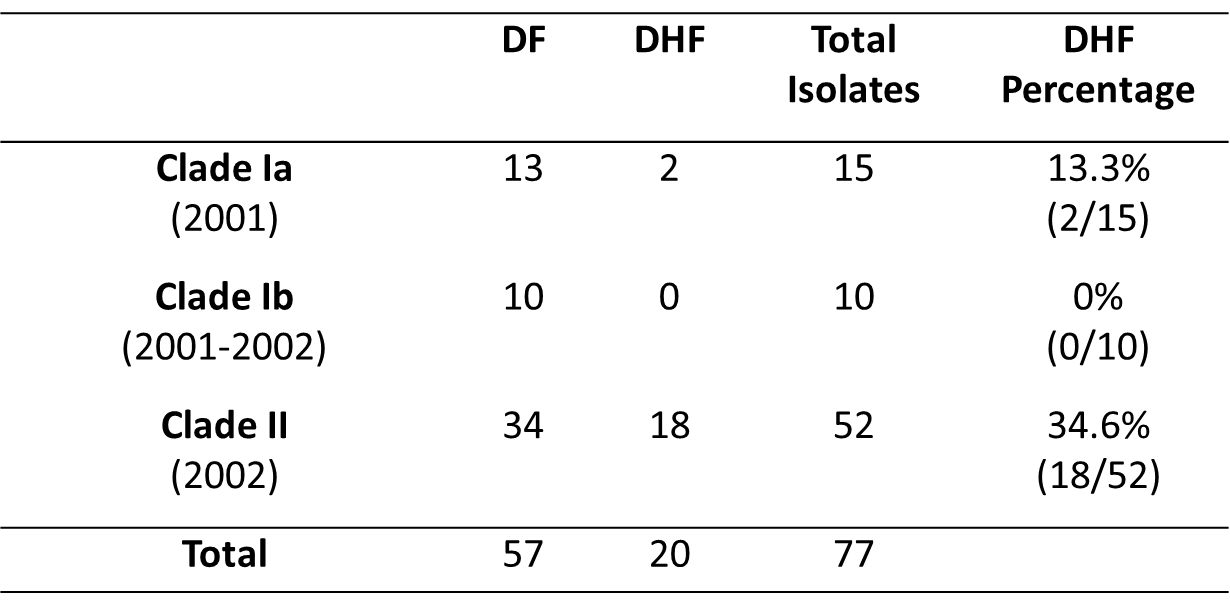


Results showed that group Ib had significantly lower percentage of DHF than group II

(p<0.05) by Fisher's exact test.
