# Supplementary material for: Inter- and intra-host sequence diversity reveal the emergence of viral variants during an overwintering epidemic caused by dengue virus serotype 2 in southern Taiwan
Source: PLoS Negl Trop Dis. 2018 Oct 4;12(10):e0006827. doi: 10.1371/journal.pntd.0006827 (PMC6191158; doi:10.1371/journal.pntd.0006827)
Supplement: S6 Table — (DOCX) [file pntd.0006827.s006.docx]

**S6 Table. Information and viral sequences used in this study for the 2001-2003 epidemics**

| **Virus strains** | **DF/ DHF** | **Gender** | **Age** | **Sampling date** | **No. of days of illness** | **Primary/ Secondary*** | **Group of viruses** |
| --- | --- | --- | --- | --- | --- | --- | --- |
| DEN0904 | DHF | M | 50 | 2001/10/31 | 2 | s | Ia |
| DEN0912 | DF | M | 38 | 2001/10/31 | 4 | s | Ib |
| DEN0915 | DF | F | 62 | 2001/11/3 | 2 | s | Ib |
| DEN0950 | DF | M | 56 | 2001/11/12 | 2 | s | Ia |
| DEN1008 | DF | F | 69 | 2001/11/28 | 2 | s | Ia |
| DEN1018 | DF | M | 14 | 2001/12/3 | 2 | p | Ia |
| DEN1019 | DF | M | 59 | 2001/12/3 | 3 | s | Ia |
| DEN1022 | DF | M | 36 | 2001/12/10 | 1 | p | Ia |
| DEN1024 | DHF | M | 56 | 2001/12/7 | 1 | p | Ia |
| DEN1025 | DF | M | 56 | 2001/12/8 | 2 | p | Ia |
| DEN1029 | DF | M | 60 | 2001/12/11 | 1 | s | Ia |
| DEN1030 | DF | F | 60 | 2001/12/12 | 1 | s | Ib |
| DEN1034 | DF | F | 53 | 2001/12/14 | 2 | p | Ib |
| DEN1039 | DF | F | 35 | 2001/12/17 | 3 | p | Ia |
| DEN1049 | DF | F | 56 | 2001/12/24 | 2 | s | Ia |
| DEN1052 | DF | F | 51 | 2001/12/22 | 3 | s | Ia |
| DEN1054 | DF | M | 42 | 2001/12/24 | 5 | s | Ia |
| DEN1060 | DF | F | 35 | 2001/12/26 | 3 | s | Ia |
| DEN1066 | DF | F | 62 | 2001/12/27 | 2 | s | Ia |
| DEN1180 | DF | F | 21 | 2002/6/16 | 4 | s | II |
| DEN1183 | DHF | F | 22 | 2002/6/17 | 4 | s | II |
| DEN1185 | DF | F | 48 | 2002/6/17 | 4 | s | Ib |
| DEN1189 | DF | F | 50 | 2002/6/18 | 0 | p | II |
| DEN1202 | DHF | F | 23 | 2002/6/22 | 2 | s | II |
| DEN1203^#^ | DF | F | 50 | 2002/6/22 | 2 | s | II |
| DEN1222^#^ | DF | F | 50 | 2002/6/27 | 7 | s | II |
| DEN1231 | DF | F | 70 | 2002/6/28 | 2 | s | II |
| DEN1246 | DF | F | 47 | 2002/6/29 | 4 | s | II |
| DEN1252 | DF | F | 48 | 2002/7/1 | 4 | s | Ib |
| DEN1272 | DF | F | 54 | 2002/7/4 | 3 | p | II |
| DEN1275 | DF | F | 82 | 2002/7/5 | 1 | s | Ib |
| DEN1280 | DF | F | 22 | 2002/7/5 | 1 | p | II |
| DEN1299 | DF | F | 60 | 2002/7/5 | 2 | s | II |

**S6 Table. Information and viral sequences analyzed in this study during the 2001-2003 epidemics (Cont’d.)**

| **Virus strains** | **DF/ DHF** | **Gender** | **Age** | **Sampling date** | **No. of days of illness** | **Primary/ Secondary*** | **Group of viruses** |
| --- | --- | --- | --- | --- | --- | --- | --- |
| DEN1315 | DF | F | 64 | 2002/7/7 | 0 | p | II |
| DEN1335 | DHF | F | 55 | 2002/7/8 | 4 | s | II |
| DEN1355 | DF | M | 20 | 2002/7/10 | 1 | p | II |
| DEN1364 | DHF | M | 61 | 2002/7/11 | 4 | s | II |
| DEN1372 | DF | M | 54 | 2002/7/11 | 5 | s | II |
| DEN1375 | DF | M | 70 | 2002/7/12 | 0 | s | Ib |
| DEN1409 | DF | F | 63 | 2002/7/16 | 3 | s | II |
| DEN1421 | DF | M | 54 | 2002/7/16 | 4 | s | II |
| DEN1439 | DHF | M | 46 | 2002/7/18 | 1 | s | II |
| DEN1461 | DF | M | 64 | 2002/7/18 | 1 | s | Ib |
| DEN1464 | DHF | M | 59 | 2002/7/20 | 2 | s | II |
| DEN1600 | DHF | M | 28 | 2002/7/28 | 3 | s | II |
| DEN1615 | DF | F | 51 | 2002/7/27 | 1 | s | Ib |
| DEN1715 | DF | F | 51 | 2002/8/4 | 2 | p | II |
| DEN1721 | DF | F | 60 | 2002/8/3 | 1 | s | II |
| DEN1852 | DF | F | 43 | 2002/8/12 | 1 | s | II |
| DEN1905 | DF | F | 61 | 2002/8/16 | 1 | s | II |
| DEN1925 | DF | F | 42 | 2002/8/16 | 5 | s | II |
| DEN1945 | DF | F | 61 | 2002/8/18 | 3 | s | II |
| DEN1949 | DF | F | 67 | 2002/8/19 | 1 | p | II |
| DEN2038 | DHF | M | 51 | 2002/8/28 | 2 | p | II |
| DEN2106 | DF | M | 58 | 2002/9/4 | 3 | s | II |
| DEN2130 | DF | M | 67 | 2002/9/9 | 4 | p | II |
| DEN2132 | DHF | F | 61 | 2002/9/9 | 4 | s | II |
| DEN2191 | DF | M | 50 | 2002/9/12 | 2 | s | II |
| DEN2208 | DHF | F | 67 | 2002/9/13 | 5 | NT | II |
| DEN2237 | DHF | M | 14 | 2002/9/14 | 8 | s | II |
| DEN2350 | DF | F | 50 | 2002/9/26 | 1 | p | II |
| DEN2419 | DF | M | 72 | 2002/10/3 | 6 | s | II |
| DEN2457 | DHF | M | 54 | 2002/10/8 | 5 | NT | II |
| DEN2533 | DHF | F | 41 | 2002/10/21 | 4 | s | II |
| DEN2534 | DF | M | 40 | 2002/10/21 | 4 | s | II |
| DEN2559 | DHF | M | 62 | 2002/10/23 | 2 | s | II |

**S6 Table. Information and viral sequences analyzed in this study during the 2001-2003 epidemics (Cont’d.)**

| **Virus strains** | **DF/ DHF** | **Gender** | **Age** | **Sampling date** | **No. of days of illness** | **Primary/ Secondary*** | **Group of viruses** |
| --- | --- | --- | --- | --- | --- | --- | --- |
| DEN2574 | DF | M | 52 | 2002/10/25 | 3 | s | II |
| DEN2584 | DF | M | 52 | 2002/10/26 | 4 | s | II |
| DEN2587 | DF | M | 41 | 2002/10/26 | 2 | s | II |
| DEN2592 | DF | M | 19 | 2002/10/26 | 1 | s | II |
| DEN2659 | DHF | M | 42 | 2002/11/1 | 3 | s | II |
| DEN2691 | DF | M | 25 | 2002/11/6 | 5 | s | II |
| DEN2747^$^ | DF | F | 42 | 2002/11/13 | 4 | s | II |
| DEN2784^$^ | DF | M | 48 | 2002/11/18 | 3 | s | II |
| DEN2992 | DHF | M | 28 | 2002/12/11 | 3 | s | II |
| DEN3011 | DF | M | 59 | 2002/12/18 | 2 | s | II |
| DEN3012 | DHF | F | 57 | 2002/12/22 | 1 | s | II |

* Primary (p) or secondary (s) infection; NT, not tested.

^#^ DEN1203 and DEN1222 are repeated samples isolated from the same patient at the different day of illness.

^$^ DEN2574 and DEN2584 are repeated sample isolated from the same patient at the different day of illness.
