# Supplementary material for: Inter- and intra-host sequence diversity reveal the emergence of viral variants during an overwintering epidemic caused by dengue virus serotype 2 in southern Taiwan
Source: PLoS Negl Trop Dis. 2018 Oct 4;12(10):e0006827. doi: 10.1371/journal.pntd.0006827 (PMC6191158; doi:10.1371/journal.pntd.0006827)
Supplement: S7 Table — (DOCX) [file pntd.0006827.s007.docx]

**S7 Table. Correlations between intra-host diversity, immune status of hosts and disease severity**


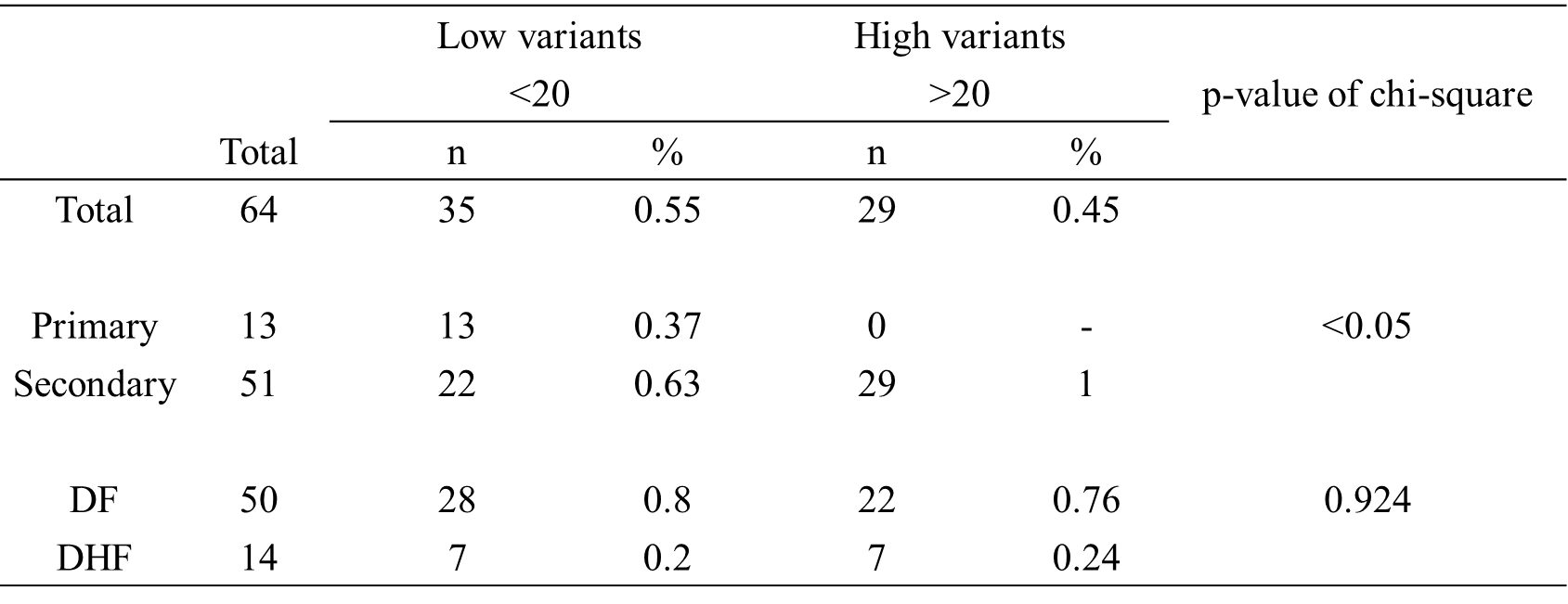


Results showed that high intra-host diversity (high variants) had significantly higher percentage of secondary infection than low variants (p<0.05). Statistics test by chi-square.
