# Supplementary material for: Inter- and intra-host sequence diversity reveal the emergence of viral variants during an overwintering epidemic caused by dengue virus serotype 2 in southern Taiwan
Source: PLoS Negl Trop Dis. 2018 Oct 4;12(10):e0006827. doi: 10.1371/journal.pntd.0006827 (PMC6191158; doi:10.1371/journal.pntd.0006827)
Supplement: S1 Fig — Maximum-likelihood tree with bootstrap values was constructed from the 73 E gene (1485 nt), using 63 DENV-2 viruses and 10 other serotypes of DENVs isolated in Taiwan and other countries. The DENV-2 viruses causing the 2001–2003 epidemic belonged to cosmopolitan genotype A and clustered with Philippine strains. (DOCX) [file pntd.0006827.s008.docx]

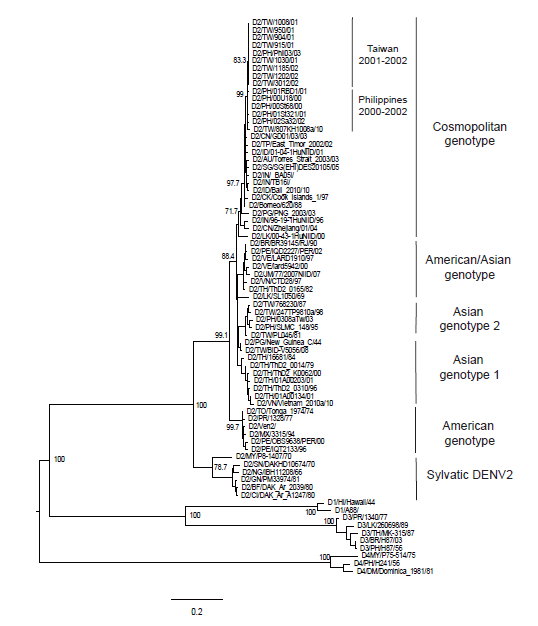


**S1 Fig. Phylogenetic analyses of DENV-2 viruses in Taiwan, 2001-2003.**

Maximum-likelihood tree with bootstrap values was constructed from the 73 E gene (1485 nt), using 63 DENV-2 viruses and 10 other serotypes of DENVs isolated in Taiwan and other countries. The DENV-2 viruses causing the 2001-2003 epidemic belonged to cosmopolitan genotype A and clustered with Philippine strains.
