# Supplementary material for: Inter- and intra-host sequence diversity reveal the emergence of viral variants during an overwintering epidemic caused by dengue virus serotype 2 in southern Taiwan
Source: PLoS Negl Trop Dis. 2018 Oct 4;12(10):e0006827. doi: 10.1371/journal.pntd.0006827 (PMC6191158; doi:10.1371/journal.pntd.0006827)
Supplement: S2 Fig — We used linear regression to analyze the genetic distance of viruses belonging to groups Ia, Ib and II based on the Open Reading Frame (ORF) sequence of each virus. Solid lines show estimated regression intervals. Group Ib has a lower evolution rate (substitutions/site/year) for the E protein compared with groups Ia and II, e.g. fewer genetic changes were observed for the E protein during the study period (Fig 5). The difference in estimated evolution rates of Ia viruses using E or ORF sequences may be due to the small sample size or that the E gene encodes a higher number of variations than the ORF gene. (DOCX) [file pntd.0006827.s009.docx]

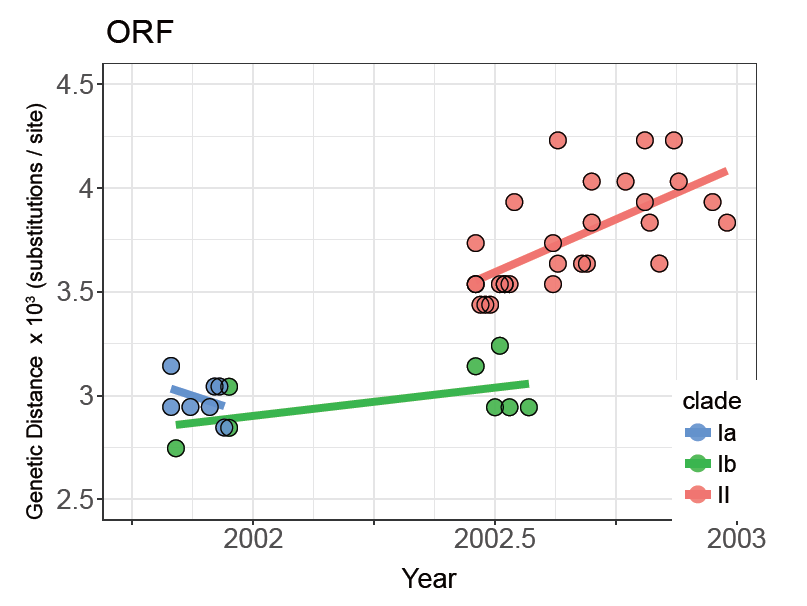


**S2 Fig. Genetic distances vs. time of the DENV-2 viruses isolated during 2001-2003 epidemics.** We used linear regression to analyze the genetic distance of viruses belonging to groups Ia, Ib and II based on the Open Reading Frame (ORF) sequence of each virus. Solid lines show estimated regression intervals. Group Ib has a lower evolution rate (substitutions/site/year) for the E protein compared with groups Ia and II, e.g. fewer genetic changes were observed for the E protein during the study period (Fig. 5). The difference in estimated evolution rates of Ia viruses using E or ORF sequences may be due to the small sample size or that the E gene encodes a higher number of variations than the ORF gene.
