# Supplementary material for: Inter- and intra-host sequence diversity reveal the emergence of viral variants during an overwintering epidemic caused by dengue virus serotype 2 in southern Taiwan
Source: PLoS Negl Trop Dis. 2018 Oct 4;12(10):e0006827. doi: 10.1371/journal.pntd.0006827 (PMC6191158; doi:10.1371/journal.pntd.0006827)
Supplement: S3 Fig — Inter-host viral genetic diversity was evaluated by modified pi, calculated by DnaSP v5 software package for the analysis of nucleotide polymorphism from aligned DNA sequence data. Intra-host viral genetic diversity was evaluated by modified pi. The three groups of viruses share different patterns (i.e. groups Ia and Ib viruses have lower genetic diversity than group II viruses) in both inter-host and intra-host diversity. Red dots indicate samples isolated in the acute phase (0–3 days after onset of illness) and blue dots indicate samples isolated in the defervescence phase (4 days after illness) with an intra-host diversity value of pi. Open diamonds indicate the inter-host diversity of the E region sequences of all three virus groups. (DOCX) [file pntd.0006827.s010.docx]

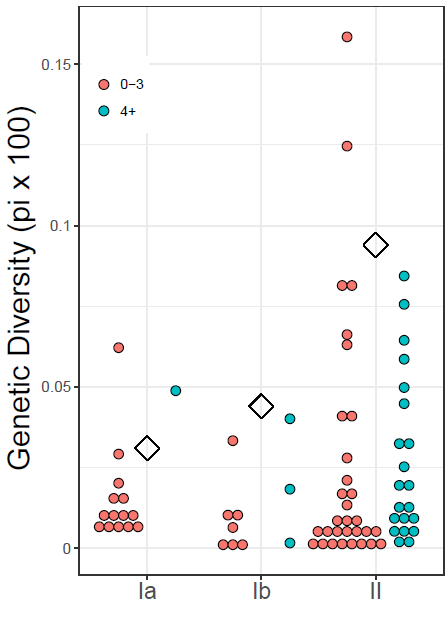


**S3 Fig. Inter-host (population) and Intra-host (individual) genetic diversity of the DENV-2 viruses isolated among Ia, Ib and II viruses.**

Inter-host viral genetic diversity was evaluated by modified pi, calculated by DnaSP v5 software package for the analysis of nucleotide polymorphism from aligned DNA sequence data [S3 Fig. Reference 1]. Intra-host viral genetic diversity was evaluated by modified pi [S3 Fig. Reference 2]. The three groups of viruses share different patterns (i.e. groups Ia and Ib viruses have lower genetic diversity than group II viruses) in both inter-host and intra-host diversity. Red dots indicate samples isolated in the acute phase (0-3 days after onset of illness) and blue dots indicate samples isolated in the defervescence phase (4 days after illness) with an intra-host diversity value of pi. Open diamonds indicate the inter-host diversity of the E region sequences of all three virus groups.

References

1. Librado P, Rozas J. DnaSP v5: a software for comprehensive analysis of DNA polymorphism data. Bioinformatics. 2009;25(11):1451-2. doi: 10.1093/bioinformatics/btp187. PubMed PMID: 19346325.

2. Leonard AS, McClain MT, Smith GJ, Wentworth DE, Halpin RA, Lin X, et al. Deep Sequencing of Influenza A Virus from a Human Challenge Study Reveals a Selective Bottleneck and Only Limited Intrahost Genetic Diversification. Journal of Virology. 2016;90(24):11247-58.
