# Supplementary material for: Inter- and intra-host sequence diversity reveal the emergence of viral variants during an overwintering epidemic caused by dengue virus serotype 2 in southern Taiwan
Source: PLoS Negl Trop Dis. 2018 Oct 4;12(10):e0006827. doi: 10.1371/journal.pntd.0006827 (PMC6191158; doi:10.1371/journal.pntd.0006827)
Supplement: S4 Fig — (A) Viral load in the group II viruses was lower than the other two groups of viruses during the acute phase (0 to 3 days after onset of illness), viral load decreased significantly after the acute phase (defervescence phase, 4–8 days after onset of illness). (B) Group Ib had significantly lower variant numbers than group Ia during the acute phase. * indicated p<0.05. Statistical analysis (two-tailed student t-test) comparing the different virus groups was completed. (DOCX) [file pntd.0006827.s011.docx]

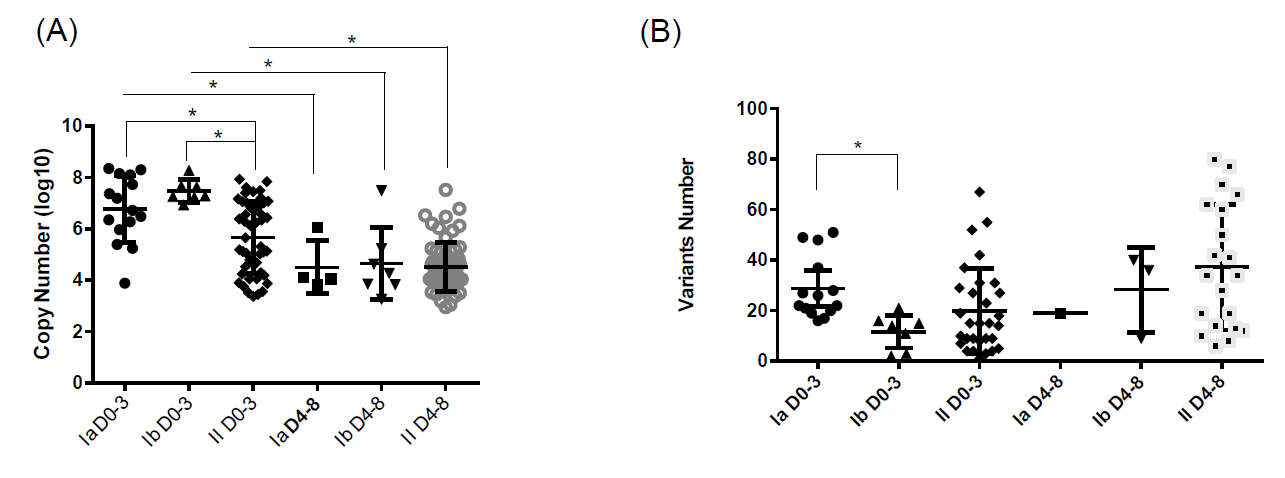


**S4 Fig. Viral loads and viral population variants found in the three virus groups, Ia, Ib and II.** (A) Viral load in the group II viruses was lower than the other two groups of viruses during the acute phase (0 to 3 days after onset of illness), viral load decreased significantly after the acute phase (defervescence phase, 4-8 days after onset of illness). (B) Group Ib had significantly lower variant numbers than group Ia during the acute phase. * indicated p<0.05. Statistical analysis (two-tailed student t-test) comparing the different virus groups was completed.
