# Supplementary material for: Inter- and intra-host sequence diversity reveal the emergence of viral variants during an overwintering epidemic caused by dengue virus serotype 2 in southern Taiwan
Source: PLoS Negl Trop Dis. 2018 Oct 4;12(10):e0006827. doi: 10.1371/journal.pntd.0006827 (PMC6191158; doi:10.1371/journal.pntd.0006827)
Supplement: S5 Fig — Bayesian Skyride plot analysis was employed and the effective population size (Ne.g) of DENV-2 is shown by a solid line with 95% highest posterior density (HPD) intervals (Shaded region) (Right axis). The bar plot indicates the number of confirmed dengue cases from 2001 to 2003 in southern Taiwan (Left axis). Effective population size based on the Bayesian method agrees well with Epi-curve analysis. (DOCX) [file pntd.0006827.s012.docx]

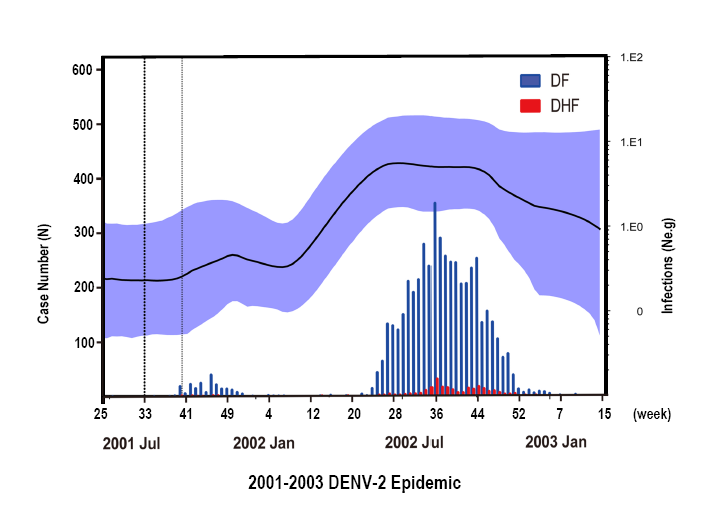


**S5 Fig. Bayesian Skyride Plot and the number of confirmed dengue cases, from July 2001 to Jan. 2003.** Bayesian Skyride plot analysis was employed and the effective population size (Ne.g) of DENV-2 is shown by a solid line with 95% highest posterior density (HPD) intervals (Shaded region) (Right axis). The bar plot indicates the number of confirmed dengue cases from 2001 to 2003 in southern Taiwan (Left axis). Effective population size based on the Bayesian method agrees well with Epi-curve analysis.
