# Supplementary material for: Inter- and intra-host sequence diversity reveal the emergence of viral variants during an overwintering epidemic caused by dengue virus serotype 2 in southern Taiwan
Source: PLoS Negl Trop Dis. 2018 Oct 4;12(10):e0006827. doi: 10.1371/journal.pntd.0006827 (PMC6191158; doi:10.1371/journal.pntd.0006827)
Supplement: S6 Fig — (A) cDNA libraries were generated by four overlapping fragments, shown as dashed lines, and were used for deep sequencing. Blue and red squares indicate the location of forward primers and reverse primers, respectively. The solid black line indicates median with gray lines showing the first (Q1) (The lowest 25% of numbers) and third (Q3) (The 75% of numbers) quartiles. (B) Distribution of variants detected by LoFreq at each nucleotide position for all analyzed samples. (C) Comparisons of median coverage versus number of variants for all sequenced samples. (DOCX) [file pntd.0006827.s013.docx]

**(A)**


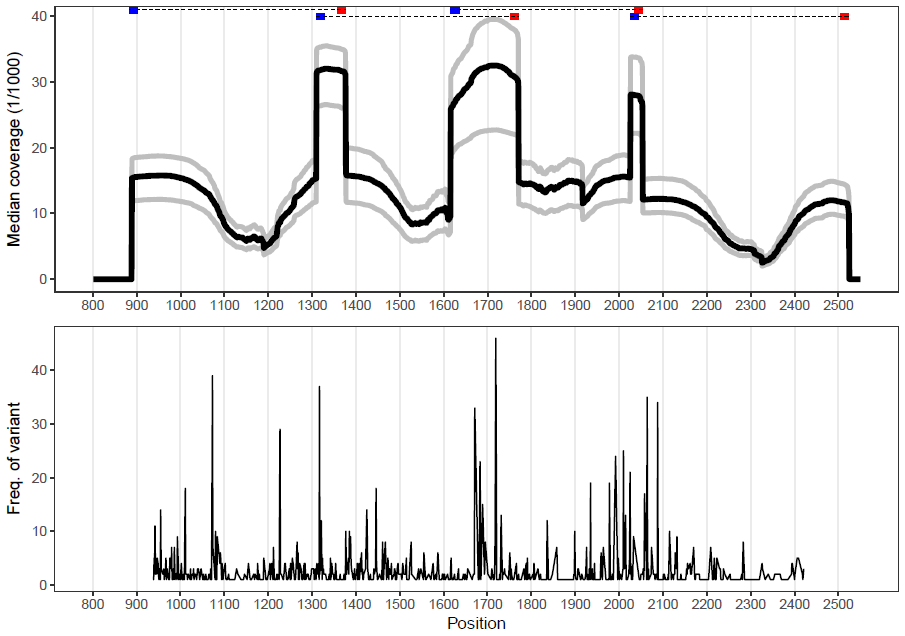


Position

**(B)**


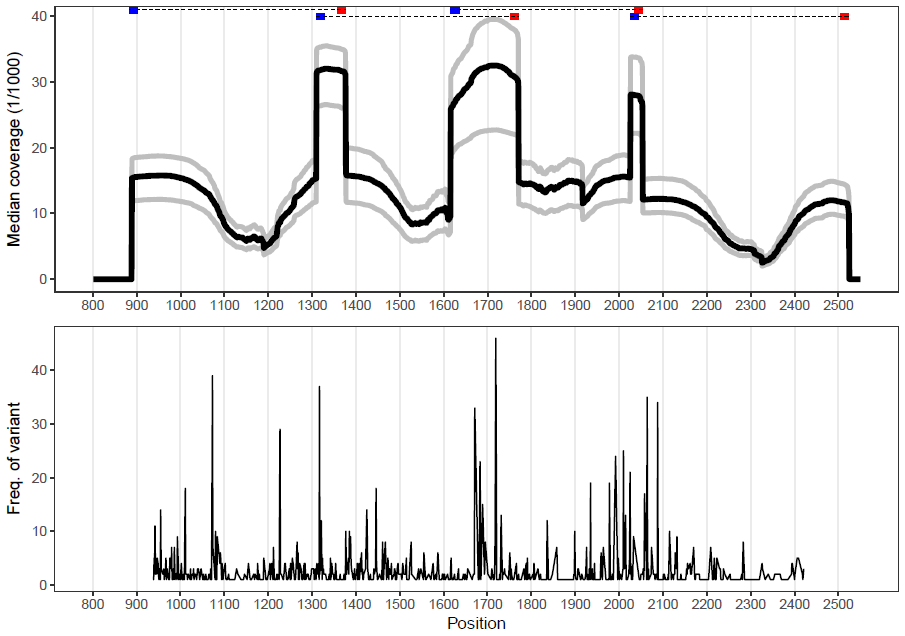


Position

**(C)**


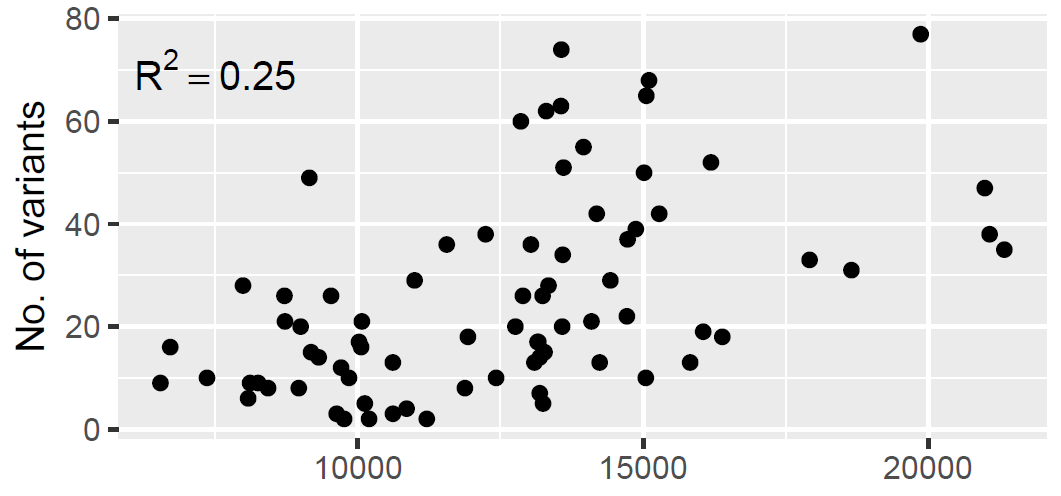


Coverage

**S6 Fig. Coverage and distribution of E gene variants identified by deep sequencing.**

(A) cDNA libraries were generated by four overlapping fragments, shown as dashed lines, and were used for deep sequencing. Blue and red squares indicate the location of forward primers and reverse primers, respectively. The solid black line indicates mediums with gray lines showing the first (Q1) (The lowest 25% of numbers) and third (Q3) (The 75% of numbers) quartiles. (B) Distribution of variants detected by LoFreq at each nucleotide position for all analyzed samples. (C) Comparisons of median coverage versus number of variants for all sequenced samples.
